# Supplementary material for: Multidimensional responses of grassland stability to eutrophication
Source: Nat Commun. 2023 Oct 11;14:6375. doi: 10.1038/s41467-023-42081-0 (PMC10567679; doi:10.1038/s41467-023-42081-0)
Supplement: Supplementary file 1 — Supplementary Information [file 41467_2023_42081_MOESM1_ESM.pdf]

Supplementary Figures

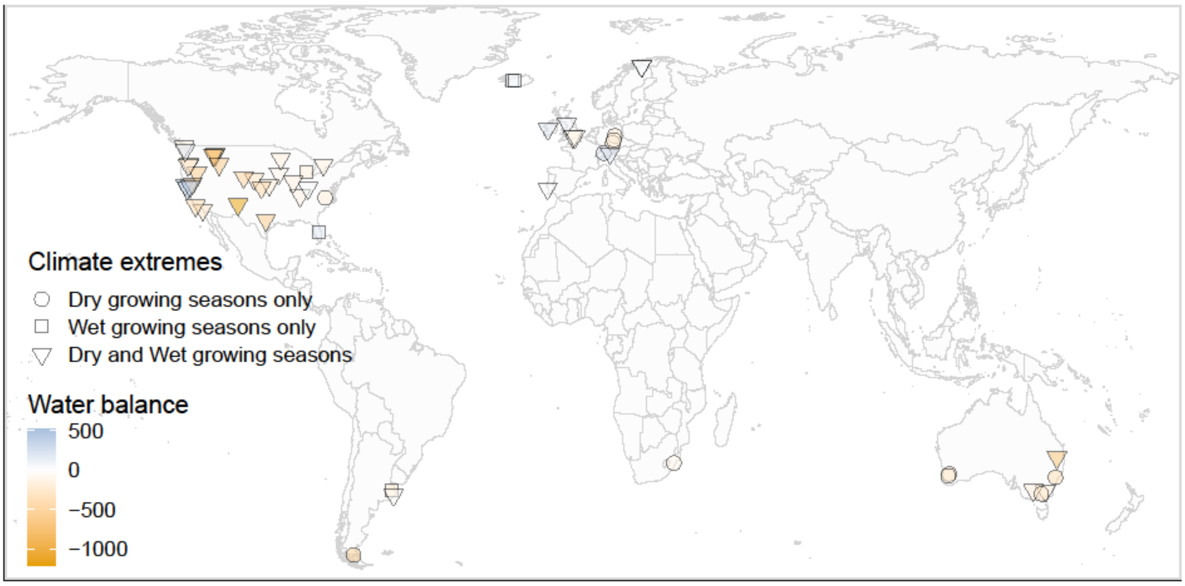

Supplementary Figure 1. Sites used in this study, their geolocation, water balance, and climate extremes. Water balance refers to the average values of precipitation minus evapotranspiration during growing seasons from 2007 to 2021. Dry/wet growing seasons only refer to sites where only dry or wet growing seasons were recorded during experimental years, dry and wet growing seasons refer to sites where both dry and wet growing seasons were recorded during experimental years. Sites are jittered to reduce overlap, see Supplementary Table 1 for more details.

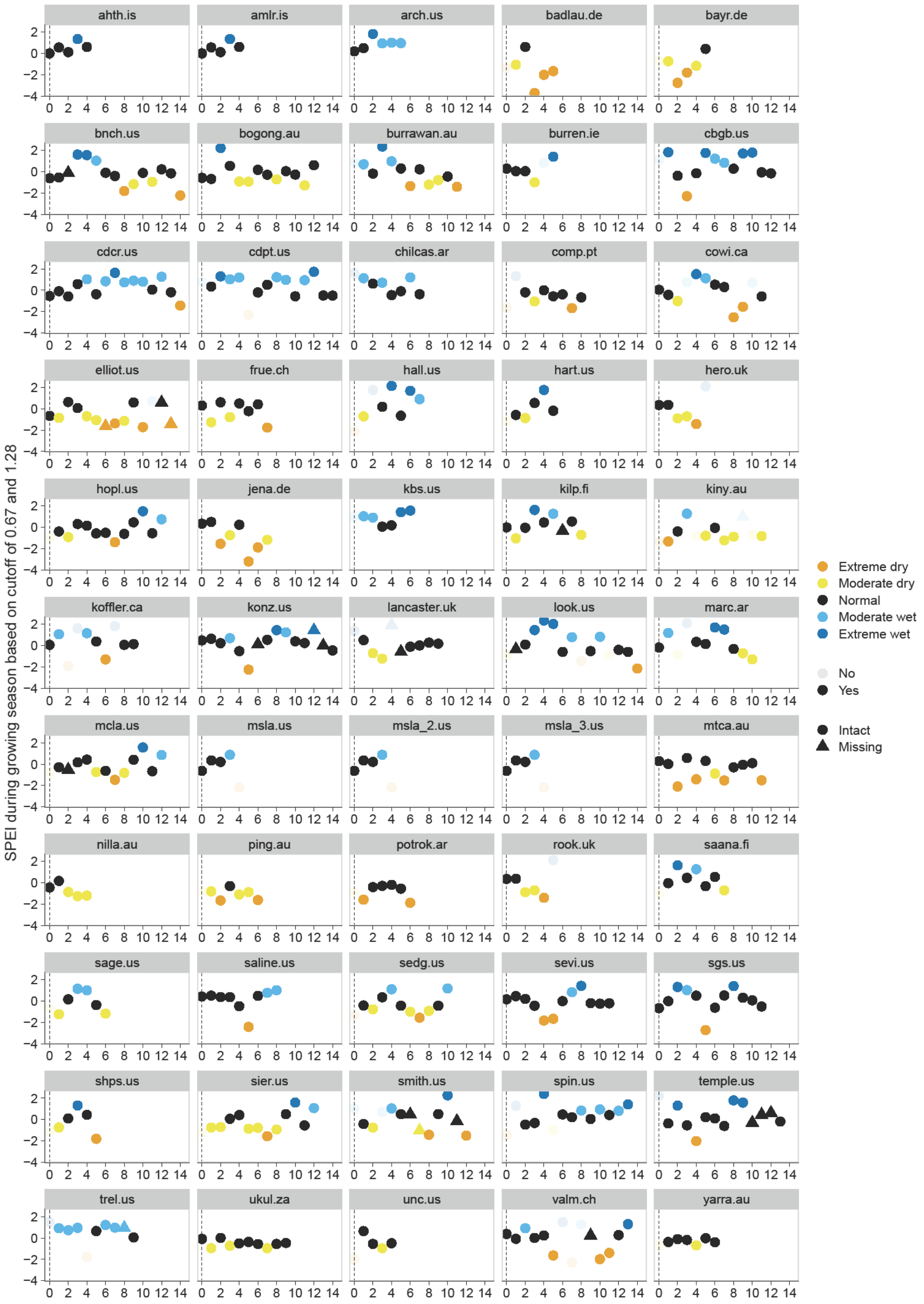

Supplementary Figure 2. Extreme dry, moderate dry, normal, moderate wet, and extreme wet growing seasons at each site during the experimental years. Positive and negative values indicate wetter and drier than normal growing seasons, respectively. SPEI accounts for water balance during the growing season based on data from January 1901 to 2021

at each site. To illustrate that dry and wet growing seasons depend on cutoffs used, at each site, we classified the treatment years (growing seasons) into extreme dry, moderate dry, normal, moderate wet, and extreme wet using the cutoff of 1.28 and 0.67 SD (1.28: occurring once per decade; 0.67: once every four years) following Isbell et al., (2015). That is, extreme dry: SPEI $\leq$ -1.28 sd; moderate dry: -1.28 sd < SPEI $\leq$ -0.67 sd; normal growing season: -0.67 sd < SPEI < 0.67 sd; moderate wet: 0.67 sd $\leq$ SPEI < 1.28 sd; and extreme wet: SPEI $\geq$ 1.28 sd. In analyses, we calculated resistance and recovery to dry and wet growing seasons were defined based on either of the cutoffs. See Supplementary Table 1 for sites, the growing seasons, and experimental years used for the analyses.

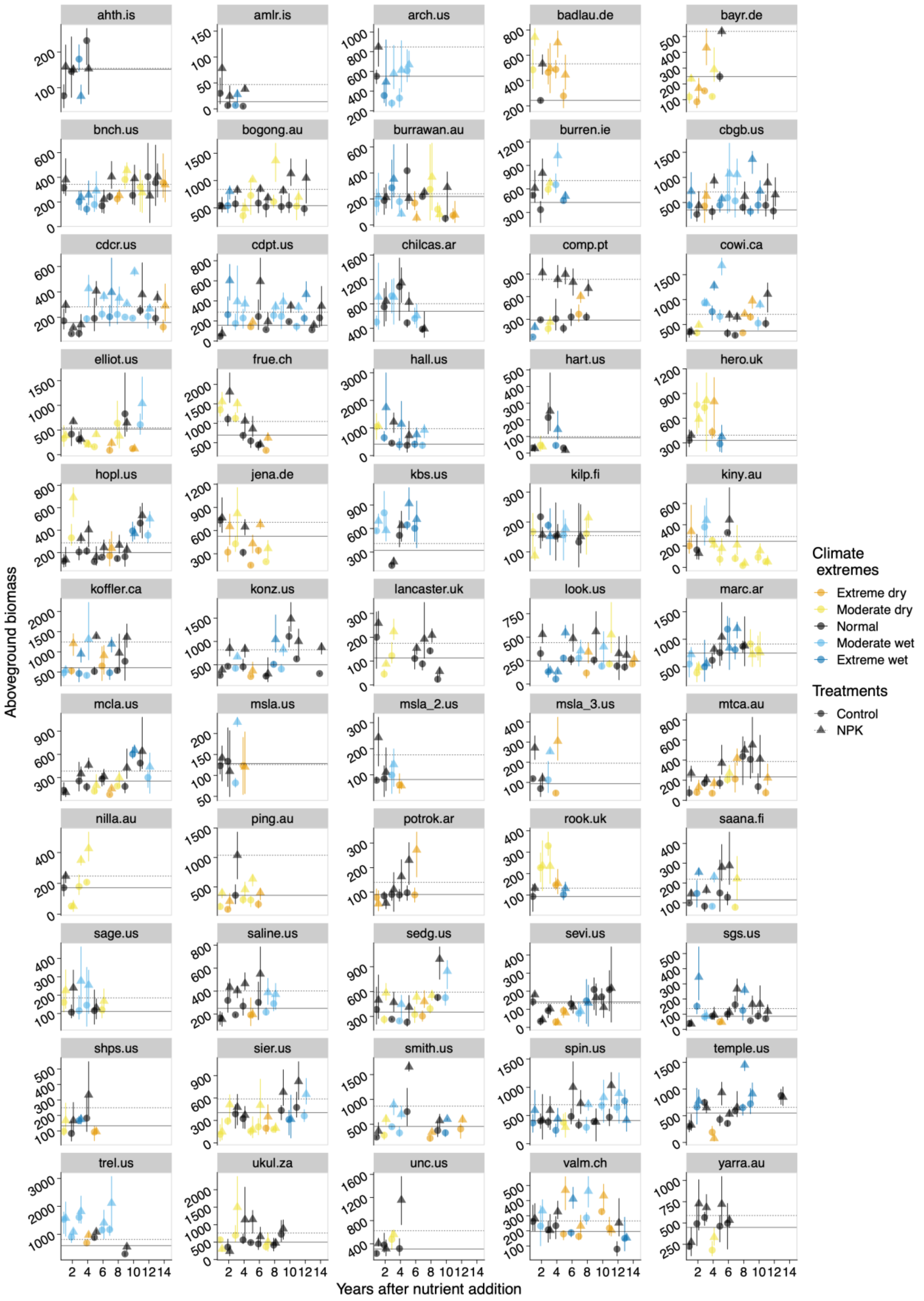

Supplementary Figure 3. Aboveground biomass (g m^{-2}) over experimental years under control and nutrient addition conditions at each site. Extreme dry, moderate dry, normal, moderate wet, and extreme wet using the cutoff of 1.28 and 0.67 SD (1.28: occurring once per decade; 0.67: once every four years) correspond to Supplementary Figure 2. Dots indicate raw

live biomass data averaged over three blocks in each year at each site. Error bars are 95% bootstrapped confidence intervals. Solid and dashed lines represent average aboveground biomass in the control and nutrient addition conditions, respectively.

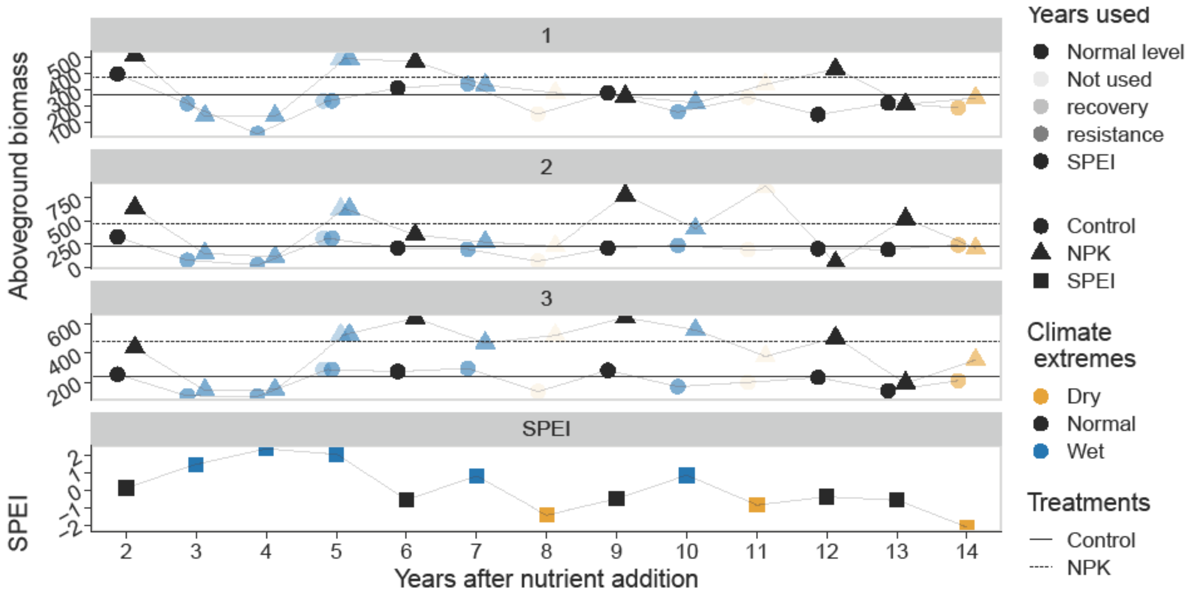

Supplementary Figure 4. Aboveground biomass (g m^{-2}) over experimental years under control and nutrient addition conditions in three blocks (shown in three panels) at site Look.us. Dry, normal, and wet growing seasons were categorized using the cutoff of 0.67 SD (i.e. a non-normal growing season occurred once every four years). Therefore, we considered both moderate and extreme dry growing seasons (from Supplementary Figure 2) as dry growing seasons, and moderate and extreme wet growing seasons (from Supplementary Figure 2) as wet growing seasons. This figure illustrates that the normal levels were calculated for each treatment in each block at each site. This figure also shows years used for the calculating resistance and recovery at site Look.us.

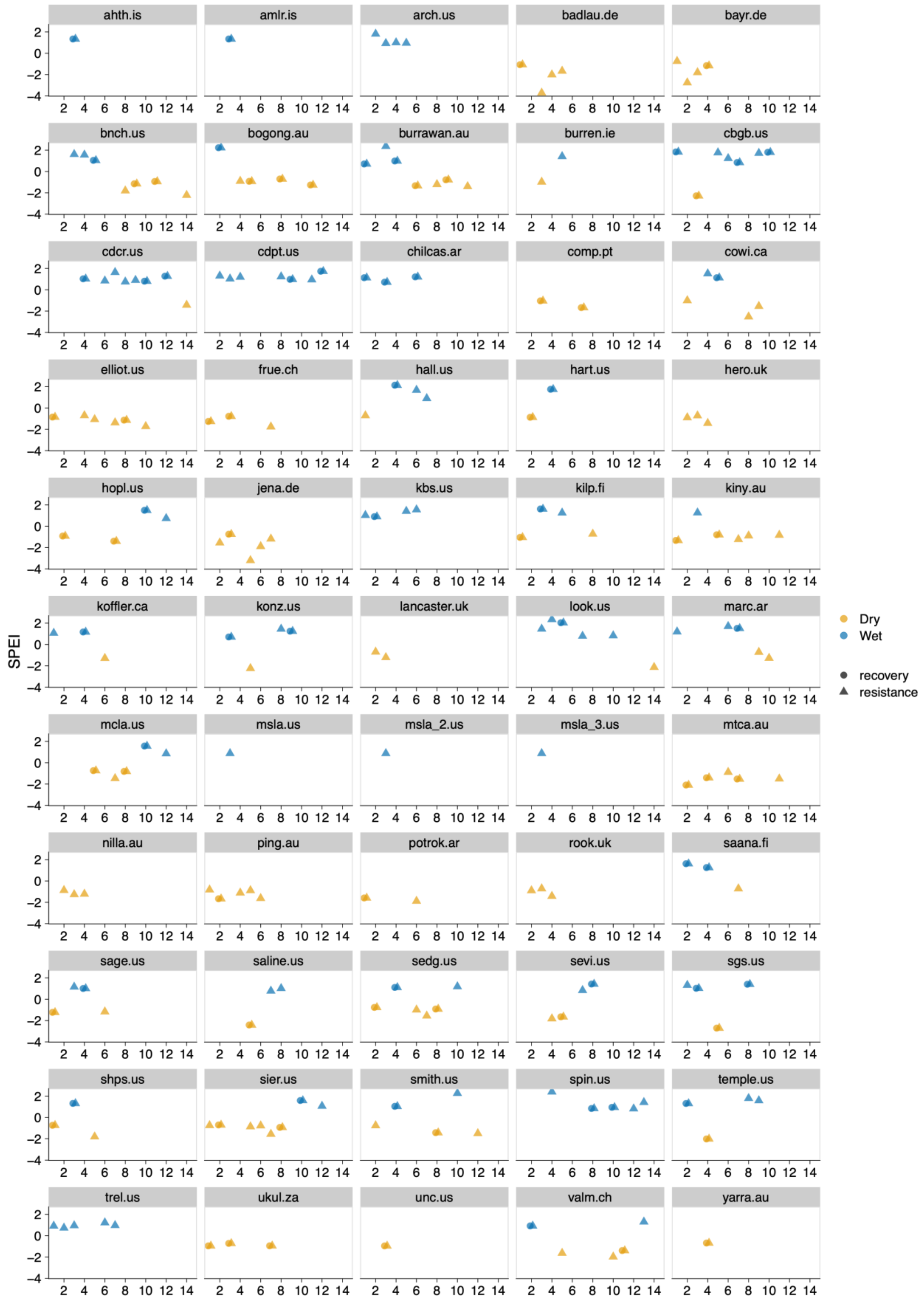

Supplementary Figure 5. Dry and wet growing seasons at each site during the experimental years used for resistance and recovery. Positive and negative values indicate wetter and drier than normal growing seasons, respectively. SPEI accounts for water balance

during the growing season based on data from January 1901 to 2021 at each site. Dry and wet growing seasons are based on 25th and 75th percentiles of SPEI (cutoff of 0.67 SD). That is, we considered both moderate and extreme dry growing seasons from Supplementary Figure 2 as dry growing seasons, and moderate and extreme wet growing seasons as wet growing seasons. We also considered dry and wet growing seasons were defined based on 10th and 90th percentiles of SPEI, where only extreme dry and wet growing seasons from Supplementary Figure 2 were used for calculating resistance and recovery, moderate dry and wet growing seasons were ignored. See Supplementary Figure 15 - 17 for results based on 10th and 90th percentiles of SPEI. See Supplementary Table 2 for combinations of three consecutive growing seasons and selection of the growing seasons for calculating resistance and recovery.

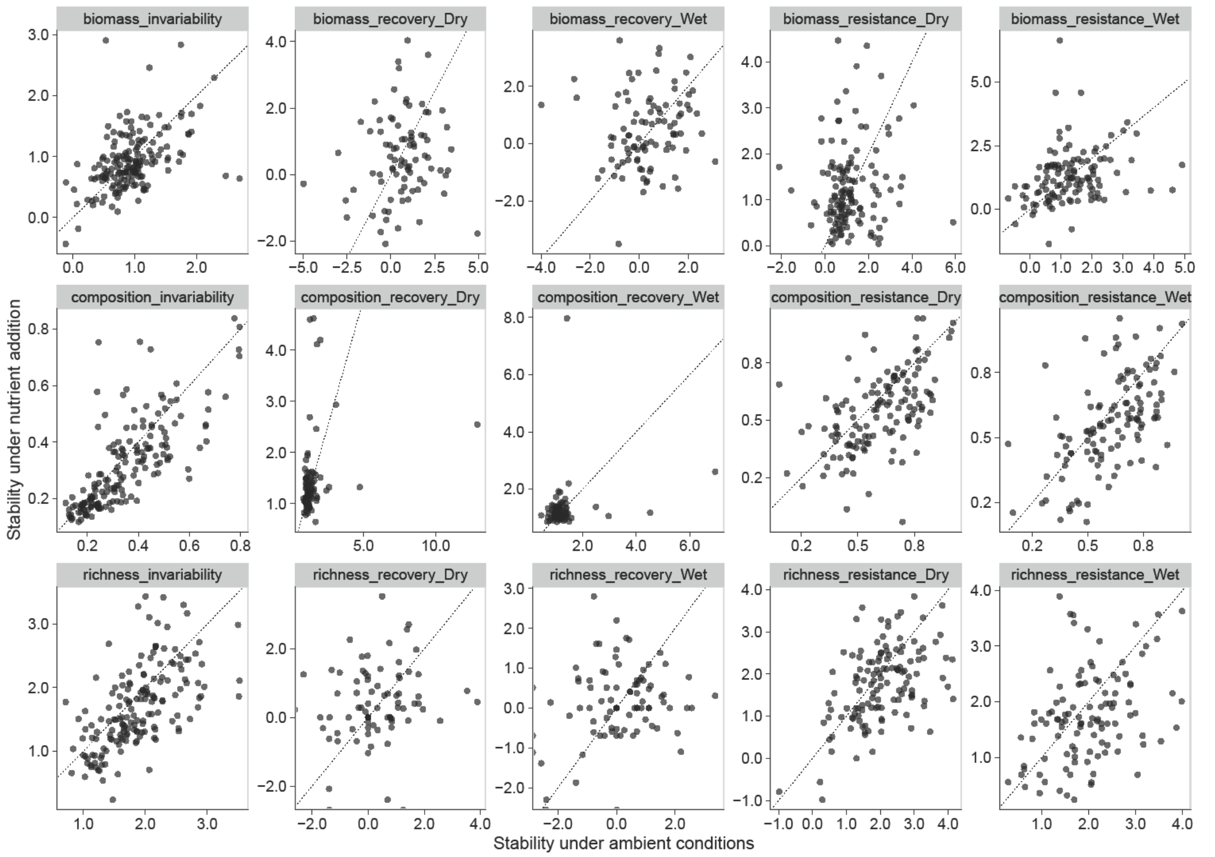

Supplementary Figure 6. Five stability facets in three community aspects under control and nutrient addition treatments at individual sites. Panel title shows stability in one community aspect for a given stability facet. Temporal invariability in biomass and species richness were calculated after detrending. Stability facets in biomass and species richness were log-transformed. Resistance and recovery for all three community aspects were averaged over years to match the data structure of temporal invariability. A dot represents one block within a site, see Supplementary Table 3 for number of sites available for different stability facets. Dotted lines represent 1:1 line, dots fall above this line indicate nutrient addition increases stability, whereas dots fall below this line indicate nutrient addition decreases stability.

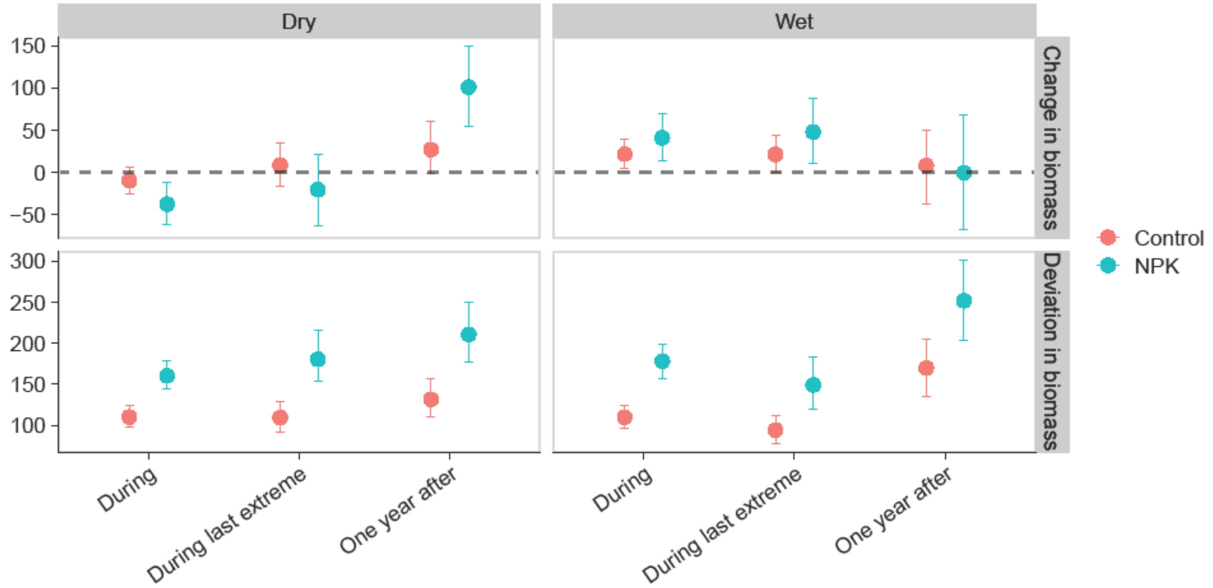

Supplementary Figure 7. Change in aboveground biomass (g m^{-2}; upper panel) and the magnitude of biomass deviation from normal levels (lower panel) under control and nutrient addition treatments. The normal levels are the means of aboveground biomass over normal growing seasons within treatments in each block at each site. During normal growing seasons, on average, nutrient addition increased aboveground biomass by 48% (control: 318.54; nutrient addition: 471.44; g.m^{-2}). Change in aboveground biomass refers to biomass differences from normal levels, thus values can be positive or negative. Deviation in aboveground biomass refers to absolute change in biomass from normal levels, thus values are positive only. “During the last extreme” refers to the last dry or wet growing season when more than one dry or wet growing seasons occur consecutively. “One year after” refers to a normal growing season after a dry or wet growing season. “During the last” and “one year after” were used to calculate recovery. Dots indicate average values over dry or wet growing seasons across sites. Error bars are 95% bootstrapped confidence intervals.

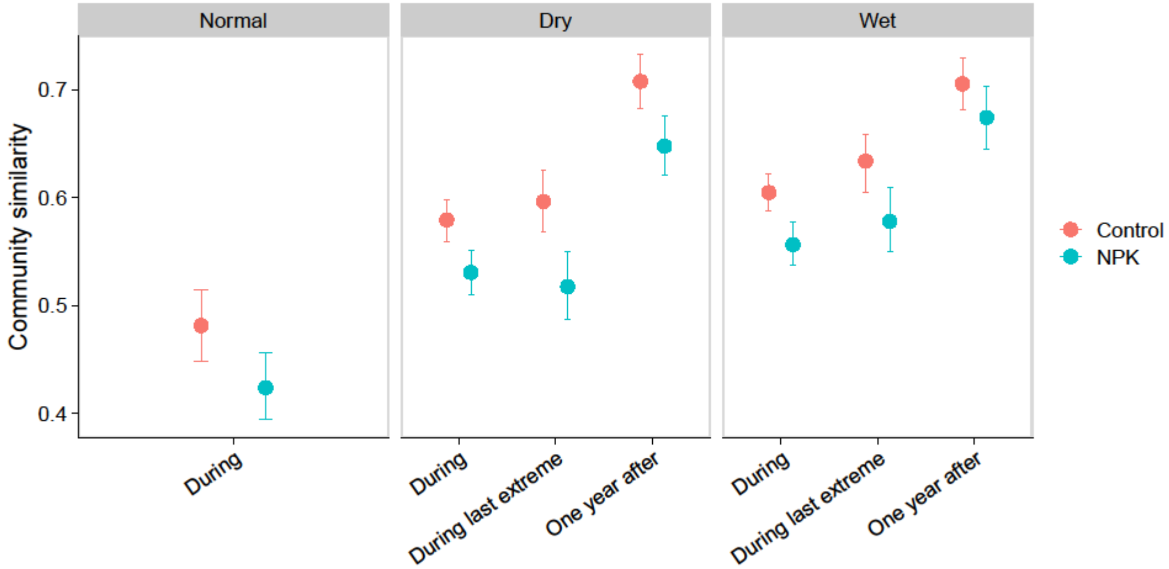

Supplementary Figure 8. Average community similarity during normal growing seasons, during and one year after dry and wet growing seasons under control and nutrient addition treatments. "During the last extreme" refers to the last dry or wet growing season when more than one dry or wet growing seasons occur consecutively. "One year after" refers to a normal growing season after a dry or wet growing season. "During the last" and "one year after" were used to calculate recovery. Dots are means, error bars are 95% bootstrapped confidence intervals.

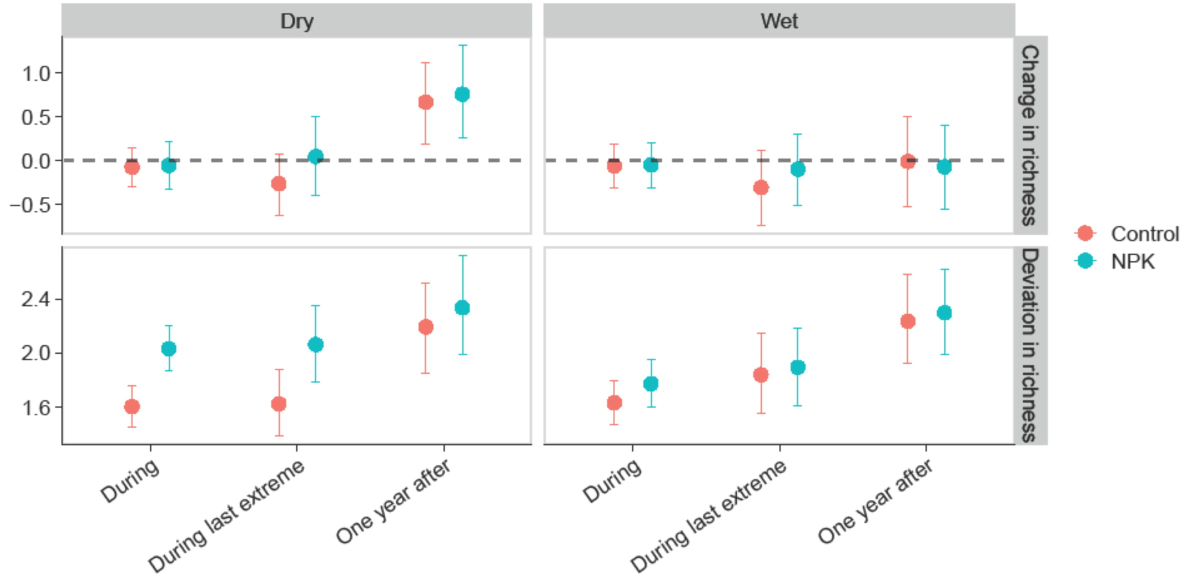

Supplementary Figure 9. Change in species richness (spp m^{-2}; upper panel) and the magnitude of richness deviation from normal levels (lower panel) under control and nutrient addition treatments. The normal levels are the means of species richness over normal growing seasons within treatments in each block at each site. During normal growing seasons, on average, nutrient addition decreased species richness by 19% (control: 12.18; nutrient addition: 9.82; spp.m^{-2}). Change in species richness refers to richness difference from normal levels, thus values can be positive or negative. Deviation in species richness refers to absolute change in richness from normal levels, thus values are positive only. “During the last extreme” refers to the last dry or wet growing season when more than one dry or wet growing seasons occur consecutively. “One year after” refers to a normal growing season after a dry or wet growing season. “During the last” and “one year after” were used to calculate recovery. Dots indicate average values during dry or wet growing seasons and across sites. Error bars and thin lines are 95% bootstrapped confidence intervals.

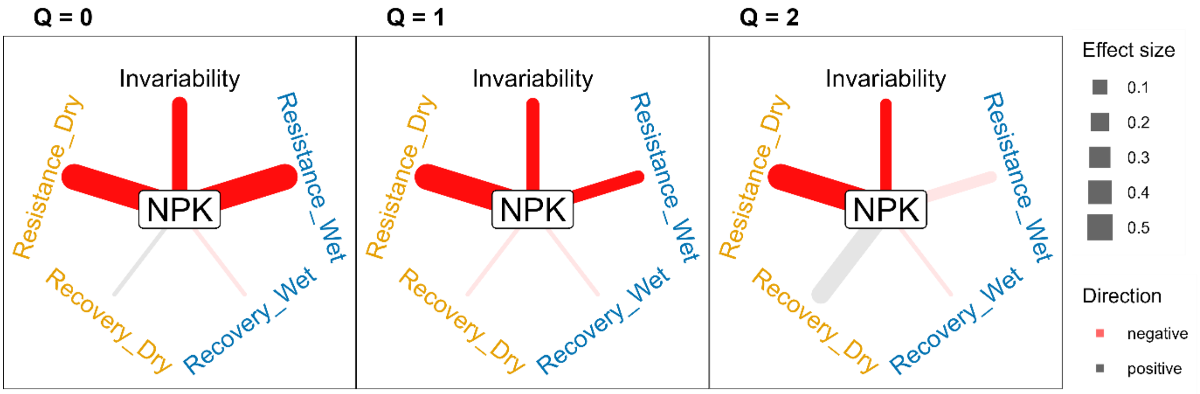

Supplementary Figure 10. Effects of nutrient addition on five facets stability in species diversity quantified as hill numbers (with varying Q). An increase in Q indicates greater weights of abundant species. Saturated line colors represent significant effects at $p \leq 0.05$, and faded line colors represent non-significant effects.

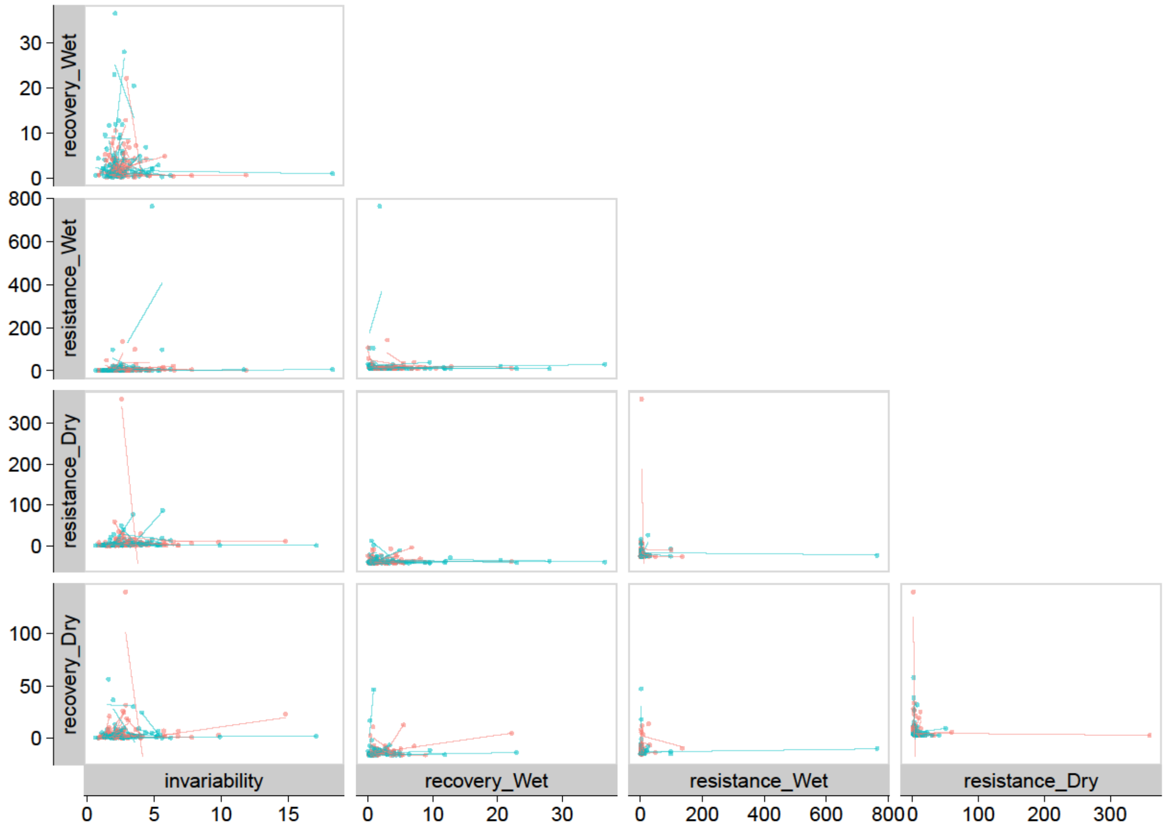

Supplementary Figure 11. Pairwise correlation among stability facets in aboveground biomass at individual sites under control (red) and nutrient addition (blue). Resistance and recovery for biomass were averaged over years to match the data structure of temporal invariability. A dot represents one block within a site. Regression lines were fitted using geom_smooth with method of "lm" from the R package ggplot to illustrate the correlation within each treatment within each site. See Supplementary Table 5 for number of sites available and overall estimate of the correlations.

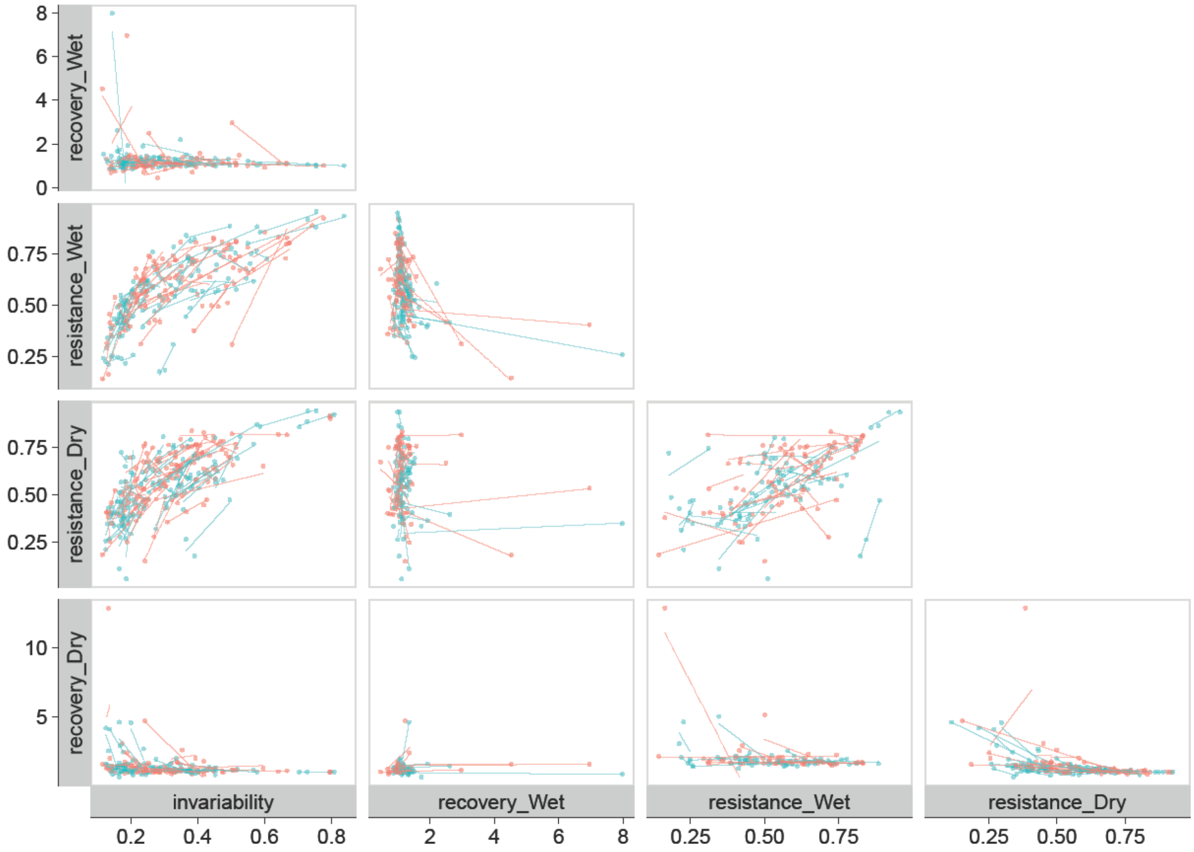

Supplementary Figure 12. Pairwise correlation among stability facets in community composition at individual sites under control (red) and nutrient addition (blue). Resistance and recovery for community composition were averaged over years to match the data structure of temporal invariability. A dot represents one block within a site. Regression lines were fitted using geom_smooth with method of "lm" from the R package ggplot to illustrate the correlation within each treatment within each site. See Supplementary Table 5 for number of sites available and overall estimate of the correlations.

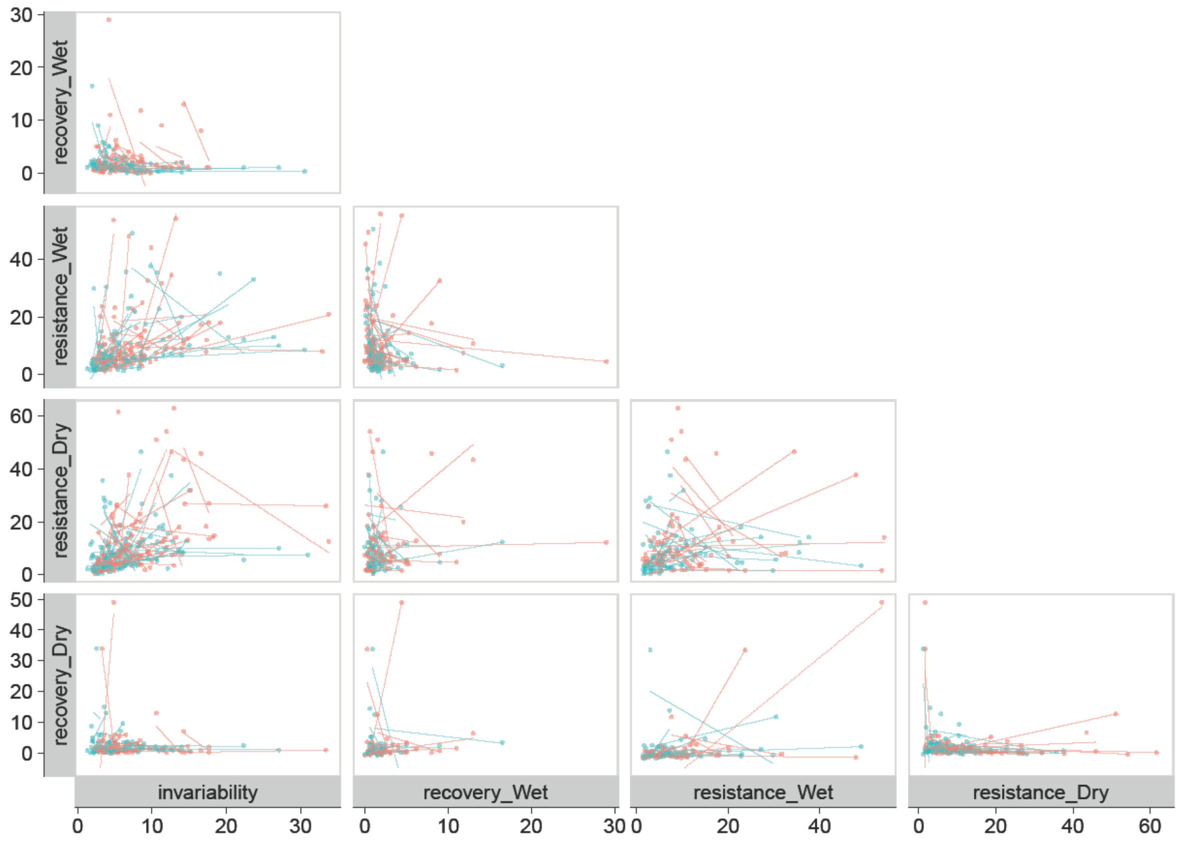

Supplementary Figure 13. Pairwise correlation among stability facets in species richness at individual sites under control (red) and nutrient addition (blue). Resistance and recovery for species richness were averaged over years to match the data structure of temporal invariability. A dot represents one block within a site. Regression lines were fitted using geom_smooth with method of "lm" from the R package ggplot to illustrate the correlation within each treatment within each site. See Supplementary Table 5 for number of sites available and overall estimate of the correlations.

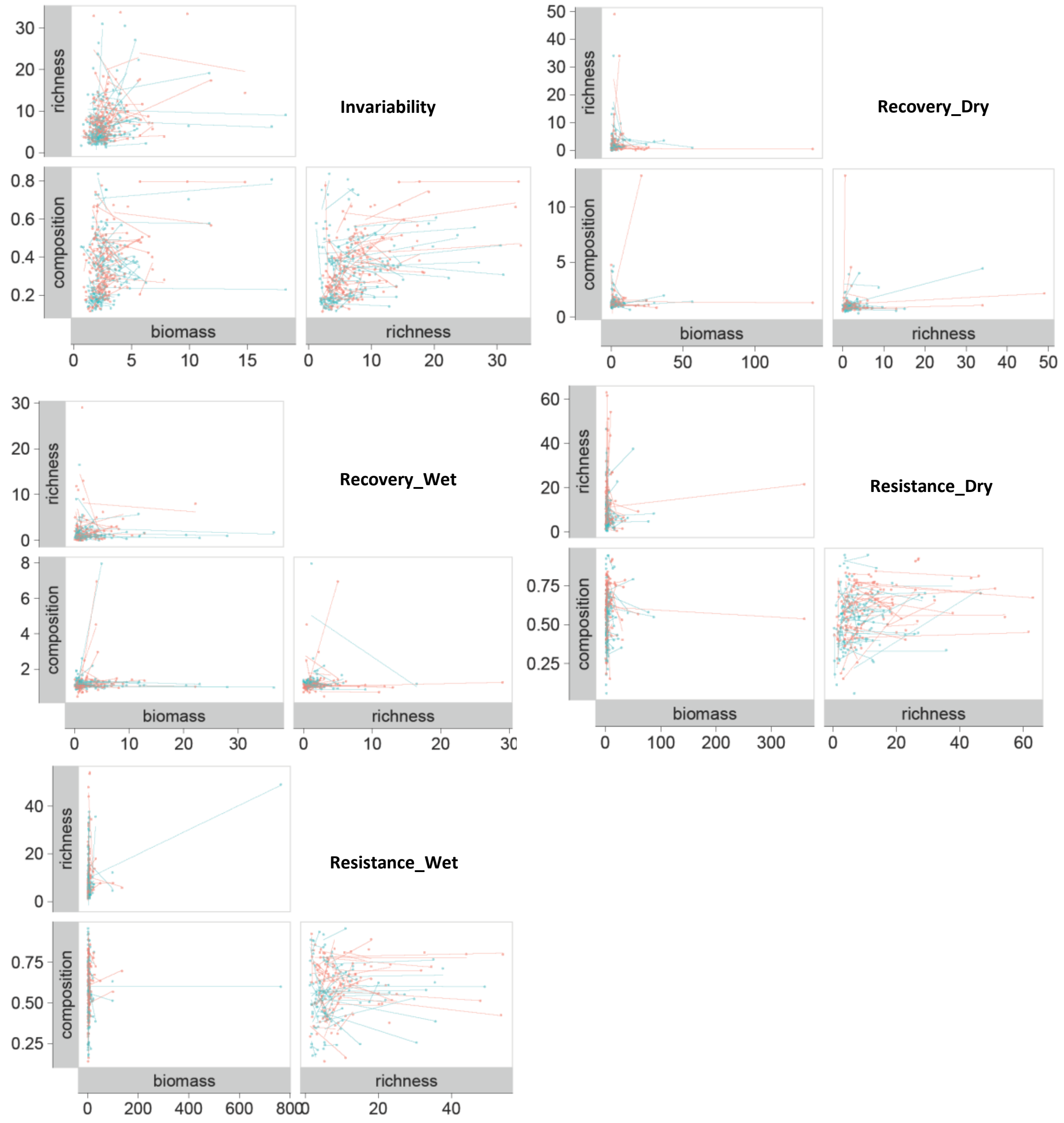

Supplementary Figure 14. Pairwise correlation among stability of three community aspects for a given stability facet at individual sites under control (red) and nutrient addition (blue). A dot represents one block within a site. Regression lines were fitted using geom_smooth with method of "lm" from the R package ggplot to illustrate the pairwise correlation within each treatment within each site. See Supplementary Table 6 for number of sites available and overall estimate of the correlations.

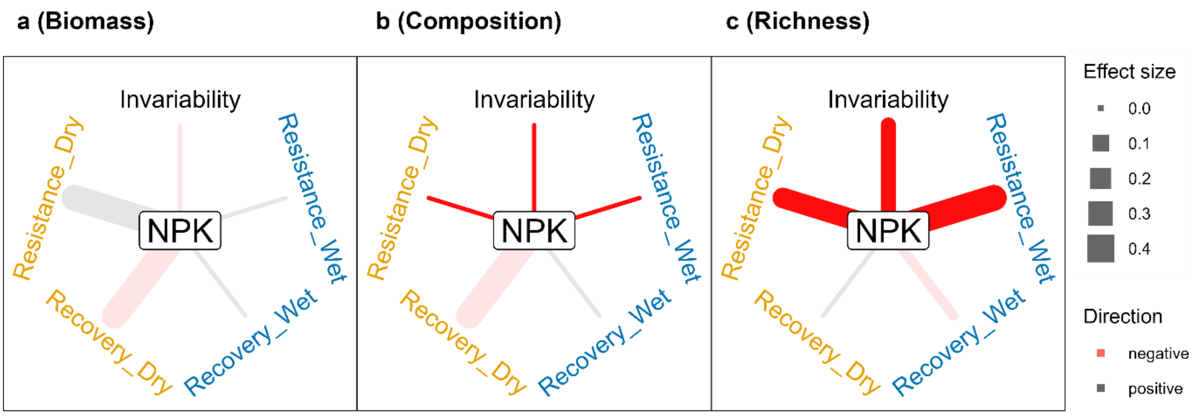

Supplementary Figure 15. Effects of nutrient addition on each of the five stability facets in each of the three community aspects. Compare with Figure 2 which used less strict definitions (25th and 75th percentiles of SPEI) of dry and wet growing seasons but more sites, here dry and wet growing seasons were defined based on 10th and 90th percentiles of SPEI. Saturated line colors represent significant effects at $p \leq 0.05$, and faded line colors represent non-significant effects.

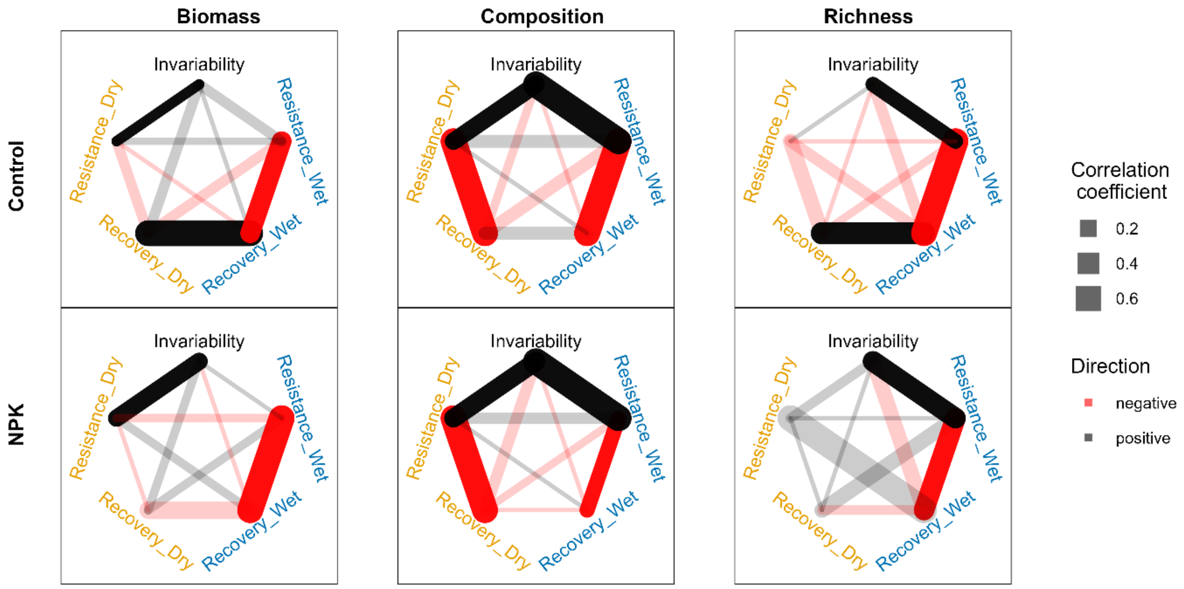

Supplementary Figure 16. Pairwise correlations among five stability facets in each of the three community aspects under control and nutrient addition conditions. Compare with Figure 3 which used less strict definitions (25th and 75th percentiles of SPEI) of dry and wet growing seasons but more sites, here dry and wet growing seasons were defined based on 10th and 90th percentiles of SPEI. Saturated line colors represent significant correlations, corresponding to 95% confidence intervals of the correlation coefficients that do not overlap with 0. Faded line colors represent non-significant correlations.

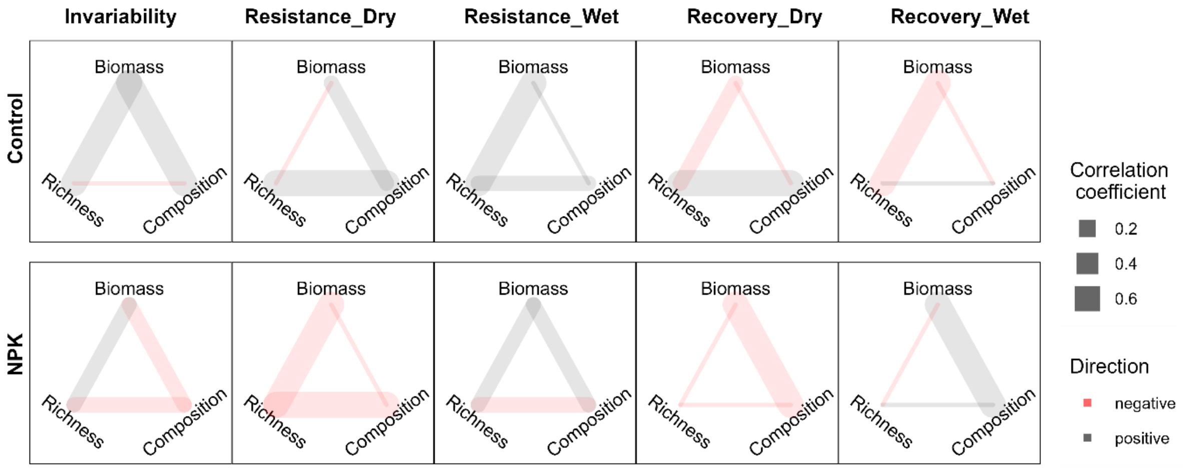

Supplementary Figure 17. Pairwise correlations among stability of the three community aspects for a given stability facet under control and nutrient addition conditions. Compare with Figure 4 which used less strict definitions (25th and 75th percentiles of SPEI) of dry and wet growing seasons but more sites, here dry and wet growing seasons were defined based on 10th and 90th percentiles of SPEI. Saturated line colors represent significant correlations, corresponding to 95% confidence intervals of the correlation coefficients that do not overlap with 0. Faded line colors represent non-significant correlations.

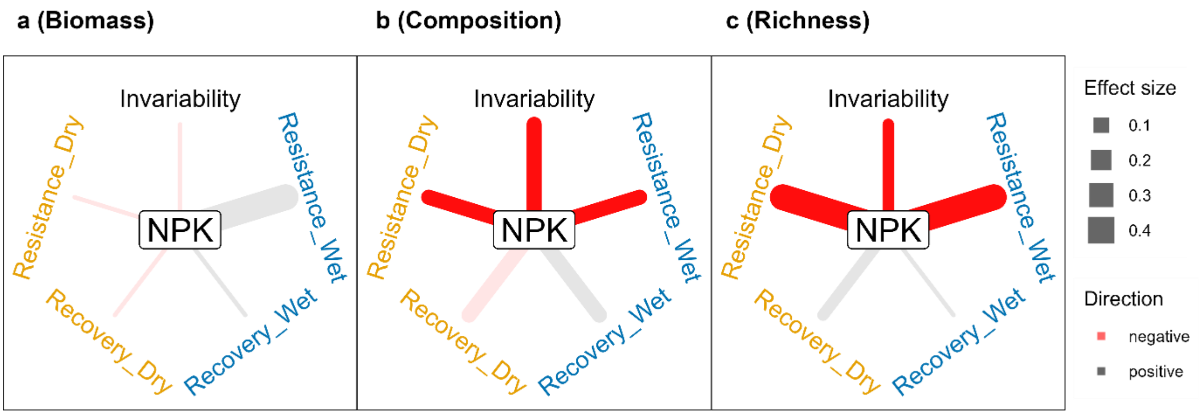

Supplementary Figure 18. Effects of nutrient addition on each of the five stability facets in each of the three community aspects. Compare with Figure 2, here dry and wet growing seasons were identified after detrending SPEI. That is, we used linear regression (function "lm") to fit SPEI over time (from 1902 to 2021), we then used the residuals from this model as the detrended SPEI. Saturated line colors represent significant effects at $p \leq 0.05$, and faded line colors represent non-significant effects.

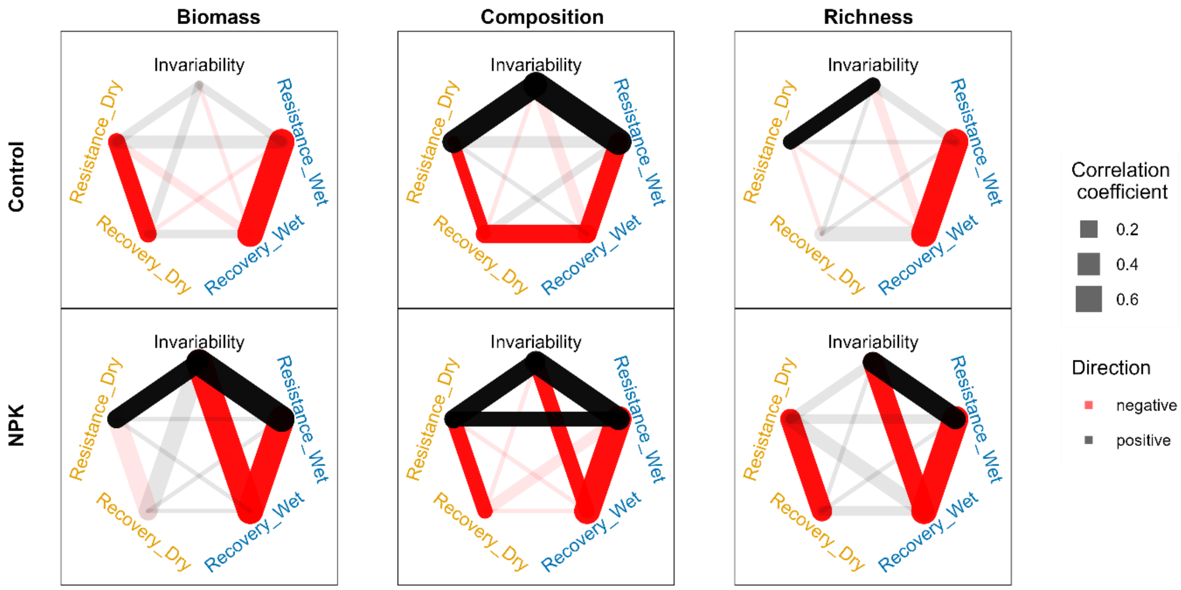

Supplementary Figure 19. Pairwise correlations among five stability facets in each of the three community aspects under control and nutrient addition conditions. Compare with Figure 2, here Dry and wet growing seasons were identified after detrending SPEI. That is, we used linear regression (function "lm") to fit SPEI over time (from 1902 to 2021), we then used the residuals from this model as the detrended SPEI. Saturated line colors represent significant correlations, corresponding to 95% confidence intervals of the correlation coefficients that do not overlap with 0. Faded line colors represent non-significant correlations.

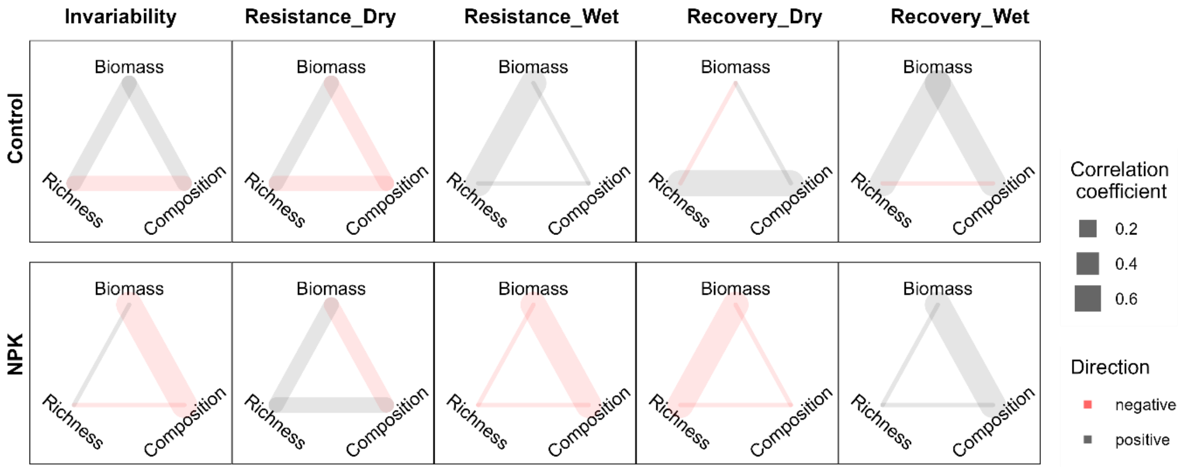

Supplementary Figure 20. Pairwise correlations among stability of the three community aspects for a given stability facet under control and nutrient addition conditions. Compare with Figure 2, here Dry and wet growing seasons were identified after detrending SPEI. That is, we used linear regression (function “lm”) to fit SPEI over time (from 1902 to 2021), we then used the residuals from this model as the detrended SPEI. Saturated line colors represent significant correlations, corresponding to 95% confidence intervals of the correlation coefficients that do not overlap with 0. Faded line colors represent non-significant correlations.

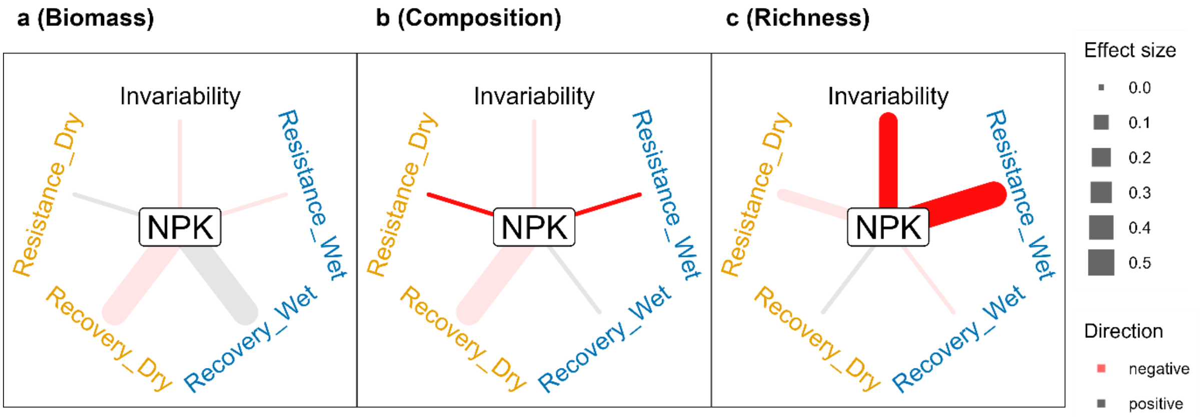

Supplementary Figure 21. Effects of nutrient addition on each of the five stability facets in each of the three community aspects. Compare with Figure 2 which used 55 sites with experimental years ranging from 4 to 15, here results were based on data from 22 sites with experimental years ranging from 10 to 15. Saturated line colors represent significant effects at $p \leq 0.05$, and faded line colors represent non-significant effects.

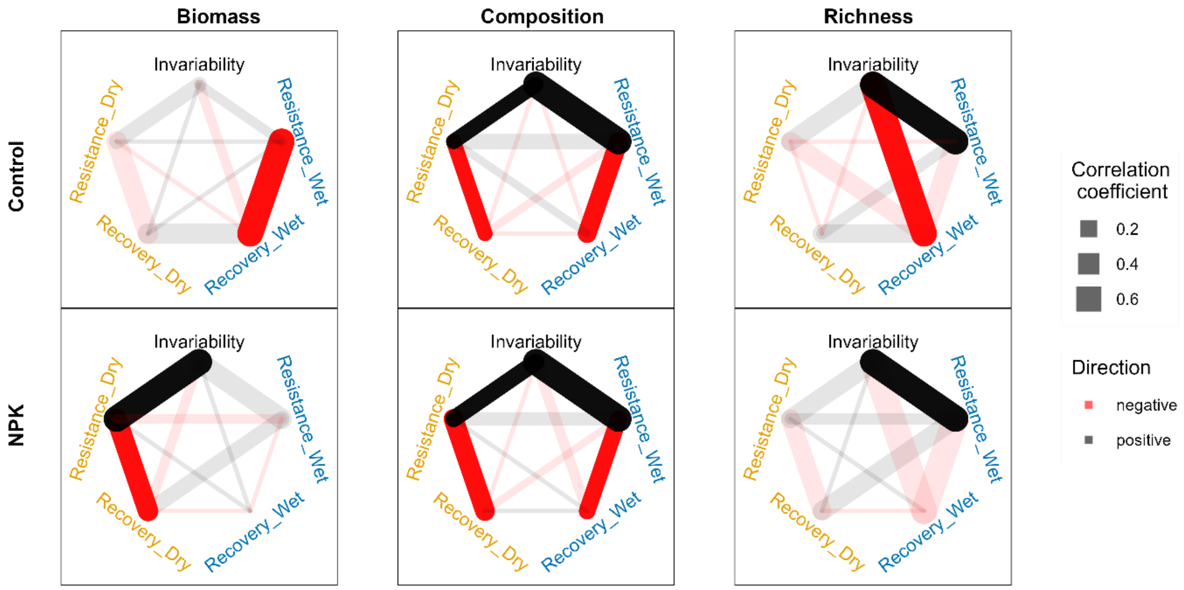

Supplementary Figure 22. Pairwise correlations among five stability facets in each of the three community aspects under control and nutrient addition conditions. Compare with Figure 3 which used 55 sites with experimental years ranging from 4 to 15, here results were based on data from 22 sites with experimental years ranging from 10 to 15. Saturated line colors represent significant correlations, corresponding to 95% confidence intervals of the correlation coefficients that do not overlap with 0. Faded line colors represent non-significant correlations.

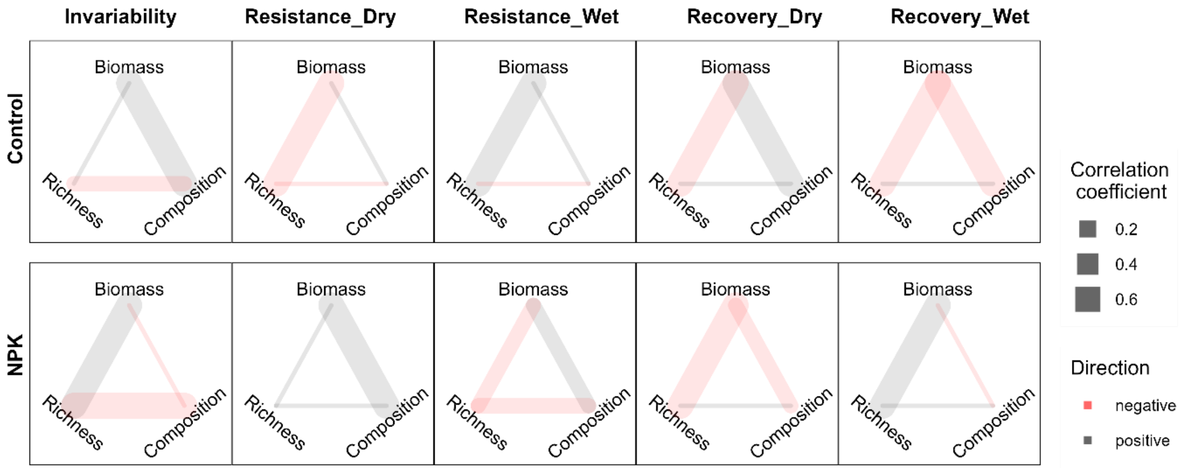

Supplementary Figure 23. Pairwise correlations among stability of the three community aspects for a given stability facet under control and nutrient addition conditions. Compare with Figure 4 which used 55 sites with experimental years ranging from 4 to 15, here results were based on data from 22 sites with experimental years ranging from 10 to 15. Saturated line colors represent significant correlations, corresponding to 95% confidence intervals of the correlation coefficients that do not overlap with 0. Faded line colors represent non-significant correlations.

Supplementary Tables

Supplementary Table 1. Information for 55 sites included in the analyses. Water balance refers to the average values of precipitation minus evapotranspiration during the growing seasons from 2007 to 2021. Growing seasons were estimated by site PIs, start and end growing season is the month when vegetation turns green and vegetation turns brown.

site_code	Habitat	Continent	Latitude	Longitude	Growing season (start to end)	Water balance (2007-2021)
ahth.is	heathland	Europe	65.13	-19.67	6-9	57.72
amlr.is	desert grassland	Europe	65.13	-19.67	6-9	57.72
arch.us	mixedgrass prairie	North America	27.17	-81.22	5-10	177.28
badlau.de	old field	Europe	51.39	11.88	4-10	-263.08
bayr.de	mesic grassland	Europe	49.92	11.58	3-9	-202.68
bnch.us	montane grassland	North America	44.28	-121.97	4-8	-410.42
bogong.au	alpine grassland	Australia	-36.87	147.25	10-1	-161.27
burrawan.au	semiarid grassland	Australia	-27.74	151.14	10-5	-768.43
burren.ie	calcareous grassland	Europe	53.07	-8.99	2-8	169.66
cbgb.us	tallgrass prairie	North America	41.79	-93.39	5-10	-121.70
cdcr.us	tallgrass prairie	North America	45.42	-93.21	4-8	-198.09
cdpt.us	shortgrass prairie	North America	41.21	-101.64	4-7	-320.66
chilcas.ar	mesic grassland	South America	-36.28	-58.27	8-3	-283.02
comp.pt	annual grassland	Europe	38.83	-8.79	10-5	26.15
cowi.ca	old field	North America	48.81	-123.63	4-7	-126.39
elliot.us	annual grassland	North America	32.88	-117.05	11-4	-320.77
frue.ch	pasture	Europe	47.11	8.54	4-9	200.73
hall.us	tallgrass prairie	North America	36.87	-86.70	4-9	-146.23
hart.us	shrub steppe	North America	42.72	-119.50	10-7	-663.66
hero.uk	mesic grassland	Europe	51.41	-0.64	4-10	-114.17
hopl.us	annual grassland	North America	39.01	-123.06	11-4	519.56
jena.de	grassland	Europe	50.94	11.53	3-10	-179.13
kbs.us	old field	North America	42.41	-85.39	4-9	-198.20
kilp.fi	tundra grassland	Europe	69.06	20.87	6-9	24.72
kiny.au	semiarid grassland	Australia	-36.20	143.75	5-10	-201.76
koffler.ca	pasture	North America	44.02	-79.54	4-8	-160.54

site_code	Habitat	Continent	Latitude	Longitude	Growing season (start to end)	Water balance (2007-2021)
konz.us	tallgrass prairie	North America	39.07	-96.58	5-9	-233.79
lancaster.uk	mesic grassland	Europe	53.99	-2.63	3-8	76.77
look.us	montane grassland	North America	44.21	-122.13	3-8	-205.99
marc.ar	grassland	South America	-37.72	-57.42	4-12	-39.35
mcla.us	annual grassland	North America	38.86	-122.41	11-4	271.61
msla.us	grassland	North America	46.66	-114.00	4-7	-389.77
msla_2.us	grassland	North America	46.66	-114.00	4-7	-779.53
msla_3.us	grassland	North America	46.66	-114.00	4-7	-779.53
mtca.au	savanna	Australia	-31.78	117.61	8-10	-261.25
nilla.au	old field	Australia	-36.90	146.01	2-1	-288.90
ping.au	old field	Australia	-32.50	116.97	4-10	-230.83
potrok.ar	semiarid grassland	South America	-51.92	-70.41	10-4	-581.11
rook.uk	mesic grassland	Europe	51.41	-0.64	4-10	-114.17
saana.fi	montane grassland	Europe	69.04	20.84	6-9	24.72
sage.us	montane grassland	North America	39.43	-120.24	4-7	-466.61
saline.us	mixedgrass prairie	North America	39.05	-99.10	5-9	-482.76
sedg.us	annual grassland	North America	34.70	-120.02	11-7	-459.86
sevi.us	desert grassland	North America	34.36	-106.69	4-11	-1214.75
sgs.us	shortgrass prairie	North America	40.82	-104.77	4-8	-565.40
shps.us	shrub steppe	North America	44.26	-112.21	4-9	-698.95
sier.us	annual grassland	North America	39.24	-121.28	11-4	415.05
smith.us	mesic grassland	North America	48.21	-122.62	10-6	130.83
spin.us	pasture	North America	38.13	-84.50	3-5	47.75
temple.us	tallgrass prairie	North America	31.04	-97.35	3-10	-656.61
trel.us	tallgrass prairie	North America	40.08	-88.83	4-9	-166.75
ukul.za	mesic grassland	Africa	-29.67	30.40	9-4	-118.91
unc.us	old field	North America	36.01	-79.02	4-9	-195.07
valm.ch	alpine grassland	Europe	46.63	10.37	6-8	176.95
yarra.au	mesic grassland	Australia	-33.61	150.74	9-3	-407.76

Supplementary Table 2. Combinations of three consecutive growing seasons and selection of the growing seasons for calculating resistance and recovery.

One year before	A given dry or wet growing season	One year after	Year for resistance	Year for recovery
Dry	Dry	Dry	Y	N
Dry	Dry	Normal	Y	Y
Dry	Dry	Wet	Y	N
Normal	Dry	Dry	Y	N
Normal	Dry	Normal	Y	Y
Normal	Dry	Wet	Y	N
Wet	Dry	Dry	N	N
Wet	Dry	Normal	N	N
Wet	Dry	Wet	N	N
Dry	Wet	Dry	N	N
Dry	Wet	Normal	N	N
Dry	Wet	Wet	N	N
Normal	Wet	Dry	Y	N
Normal	Wet	Normal	Y	Y
Normal	Wet	Wet	Y	N
Wet	Wet	Dry	Y	N
Wet	Wet	Normal	Y	Y
Wet	Wet	Wet	Y	N

Supplementary Table 3. Model summaries for the fixed effects of nutrient addition on the five stability facets in three community aspects. Models were specified as lme (stability facet ~ Treatment, random=~1|site/block). Stability facets based on aboveground biomass, species richness, but not community composition, are on the log scale. The significance of treatment effects was assessed using t-test. p: p-value for the 2-tailed test. r2m (marginal R2): proportion of variance explained by the fixed effects in the model; r2c (conditional R2): proportion of variance explained by the fixed and random effects. SD (block); standard deviation for the random effect of blocks (nested within sites); SD (site): standard deviation for the random effect of sites.

Community aspect	Stability facet	Terms	Value	Std Error	DF	t-value	p	r2m	r2c	SD (block)	SD (site)
biomass	invariability	(Intercept)	0.94	0.05	164	18.97	0.00	0.00	0.47	0.10	0.30
biomass	invariability	trtNPK	-0.02	0.04	164	-0.54	0.59	0.00	0.47	0.10	0.30
biomass	recovery_Dry	(Intercept)	0.67	0.18	83	3.70	0.00	0.00	0.21	0.00	0.64
biomass	recovery_Dry	trtNPK	-0.13	0.19	83	-0.69	0.49	0.00	0.21	0.00	0.64
biomass	recovery_Wet	(Intercept)	0.32	0.15	92	2.11	0.04	0.00	0.21	0.00	0.56
biomass	recovery_Wet	trtNPK	0.14	0.16	92	0.85	0.39	0.00	0.21	0.00	0.56
biomass	resistance_Dry	(Intercept)	1.16	0.09	131	12.37	0.00	0.00	0.11	0.00	0.32
biomass	resistance_Dry	trtNPK	0.10	0.11	131	0.87	0.39	0.00	0.11	0.00	0.32
biomass	resistance_Wet	(Intercept)	1.32	0.12	116	10.69	0.00	0.00	0.32	0.00	0.59
biomass	resistance_Wet	trtNPK	-0.02	0.11	116	-0.19	0.85	0.00	0.32	0.00	0.59
composition	recovery_Dry	(Intercept)	1.43	0.13	92	10.92	0.00	0.00	0.21	0.12	0.48
composition	recovery_Dry	trtNPK	-0.01	0.14	92	-0.10	0.92	0.00	0.21	0.12	0.48
composition	recovery_Wet	(Intercept)	1.23	0.09	92	13.93	0.00	0.00	0.19	0.18	0.28
composition	recovery_Wet	trtNPK	0.04	0.10	92	0.39	0.70	0.00	0.19	0.18	0.28
composition	resistance_Dry	(Intercept)	0.59	0.02	131	28.06	0.00	0.03	0.60	0.00	0.13
composition	resistance_Dry	trtNPK	-0.06	0.01	131	-4.30	0.00	0.03	0.60	0.00	0.13
composition	resistance_Wet	(Intercept)	0.61	0.02	116	25.83	0.00	0.02	0.63	0.00	0.13
composition	resistance_Wet	trtNPK	-0.05	0.01	116	-3.63	0.00	0.02	0.63	0.00	0.13
richness	invariability	(Intercept)	1.88	0.07	164	26.78	0.00	0.02	0.62	0.00	0.47
richness	invariability	trtNPK	-0.19	0.04	164	-4.47	0.00	0.02	0.62	0.00	0.47
richness	recovery_Dry	(Intercept)	0.32	0.14	74	2.24	0.03	0.00	0.21	0.00	0.49
richness	recovery_Dry	trtNPK	0.08	0.15	74	0.54	0.59	0.00	0.21	0.00	0.49
richness	recovery_Wet	(Intercept)	0.24	0.13	80	1.81	0.07	0.00	0.22	0.00	0.48
richness	recovery_Wet	trtNPK	-0.07	0.14	80	-0.50	0.62	0.00	0.22	0.00	0.48
richness	resistance_Dry	(Intercept)	2.04	0.11	127	18.39	0.00	0.02	0.50	0.07	0.63
richness	resistance_Dry	trtNPK	-0.28	0.08	127	-3.50	0.00	0.02	0.50	0.07	0.63
richness	resistance_Wet	(Intercept)	2.01	0.10	109	20.32	0.00	0.02	0.38	0.09	0.49
richness	resistance_Wet	trtNPK	-0.22	0.09	109	-2.53	0.01	0.02	0.38	0.09	0.49

Supplementary Table 4. Model summaries for the fixed effects of nutrient addition on normal levels, change and deviation of community aspects from their normal levels. Note, change can be positive or negative values; while deviation is always positive values. For the normal levels, models were specified as lme (normal level ~ Treatment, random=~1|site/block). For the change and deviation, models were specified as lme (change/deviation ~ Treatment, random=~1|site/block/year). Deviation of biomass and species richness, but not community composition, are on the log scale. The significance of treatment effects was assessed using t-test. p: p-value for the 2-tailed test. r2m (marginal R2): proportion of variance explained by the fixed effects in the model; r2c (conditional R2): proportion of variance explained by the fixed and random effects.

Community aspect	Value type	Events	Terms	Value	Std. Error	DF	t-value	p	r2m	r2c
biomass	Deviation	During Dry	(Intercept)	3.65	0.37	344	9.75	0.00	0.01	0.87
biomass	Deviation	During Dry	trtNPK	0.41	0.07	344	5.62	0.00	0.01	0.87
biomass	Deviation	During last Dry	(Intercept)	3.39	0.51	143	6.59	0.00	0.01	0.89
biomass	Deviation	During last Dry	trtNPK	0.46	0.12	143	3.90	0.00	0.01	0.89
biomass	Deviation	During last Wet	(Intercept)	3.34	0.52	137	6.46	0.00	0.00	0.88
biomass	Deviation	During last Wet	trtNPK	0.33	0.13	137	2.60	0.01	0.00	0.88
biomass	Deviation	During Wet	(Intercept)	3.44	0.41	308	8.30	0.00	0.01	0.85
biomass	Deviation	During Wet	trtNPK	0.45	0.09	308	5.18	0.00	0.01	0.85
biomass	Deviation	One year after Dry	(Intercept)	4.34	0.14	143	30.91	0.00	0.02	0.36
biomass	Deviation	One year after Dry	trtNPK	0.33	0.13	143	2.62	0.01	0.02	0.36
biomass	Deviation	One year after Wet	(Intercept)	4.37	0.21	131	20.77	0.00	0.01	0.46
biomass	Deviation	One year after Wet	trtNPK	0.27	0.14	131	1.84	0.07	0.01	0.46
biomass	Change	During Dry	(Intercept)	-11.69	12.87	344	-0.91	0.36	0.01	0.50
biomass	Change	During Dry	trtNPK	-27.84	10.56	344	-2.64	0.01	0.01	0.50
biomass	Change	During last Dry	(Intercept)	-6.57	26.13	143	-0.25	0.80	0.00	0.50
biomass	Change	During last Dry	trtNPK	-29.02	18.58	143	-1.56	0.12	0.00	0.50
biomass	Change	During last Wet	(Intercept)	24.43	18.55	137	1.32	0.19	0.00	0.30
biomass	Change	During last Wet	trtNPK	26.55	19.51	137	1.36	0.18	0.00	0.30
biomass	Change	During Wet	(Intercept)	22.52	15.81	308	1.42	0.16	0.00	0.30
biomass	Change	During Wet	trtNPK	19.29	14.69	308	1.31	0.19	0.00	0.30
biomass	Change	One year after Dry	(Intercept)	27.77	26.01	143	1.07	0.29	0.02	0.39
biomass	Change	One year after Dry	trtNPK	74.11	22.92	143	3.23	0.00	0.02	0.39
biomass	Change	One year after Wet	(Intercept)	5.07	49.87	131	0.10	0.92	0.00	0.62
biomass	Change	One year after Wet	trtNPK	-8.41	26.74	131	-0.31	0.75	0.00	0.62
composition	Deviation	During Dry	(Intercept)	0.59	0.02	383	30.16	0.00	0.02	0.49
composition	Deviation	During Dry	trtNPK	-0.05	0.01	383	-4.84	0.00	0.02	0.49
composition	Deviation	During last Dry	(Intercept)	0.61	0.03	143	22.74	0.00	0.04	0.59
composition	Deviation	During last Dry	trtNPK	-0.08	0.01	143	-5.55	0.00	0.04	0.59
composition	Deviation	During last Wet	(Intercept)	0.63	0.02	137	27.09	0.00	0.03	0.46
composition	Deviation	During last Wet	trtNPK	-0.06	0.02	137	-3.59	0.00	0.03	0.46
composition	Deviation	During Wet	(Intercept)	0.59	0.02	353	27.64	0.00	0.02	0.53
composition	Deviation	During Wet	trtNPK	-0.05	0.01	353	-4.97	0.00	0.02	0.53
composition	Deviation	One year after Dry	(Intercept)	0.73	0.03	140	26.63	0.00	0.03	0.74
composition	Deviation	One year after Dry	trtNPK	-0.06	0.01	140	-5.66	0.00	0.03	0.74
composition	Deviation	One year after Wet	(Intercept)	0.70	0.02	131	29.27	0.00	0.01	0.59
composition	Deviation	One year after Wet	trtNPK	-0.03	0.01	131	-2.41	0.02	0.01	0.59
richness	Deviation	During Dry	(Intercept)	-0.34	0.24	344	-1.43	0.15	0.00	0.52
richness	Deviation	During Dry	trtNPK	0.29	0.11	344	2.54	0.01	0.00	0.52
richness	Deviation	During last Dry	(Intercept)	-0.45	0.34	143	-1.35	0.18	0.01	0.60
richness	Deviation	During last Dry	trtNPK	0.33	0.17	143	1.91	0.06	0.01	0.60
richness	Deviation	During last Wet	(Intercept)	-0.46	0.33	137	-1.37	0.17	0.00	0.63
richness	Deviation	During last Wet	trtNPK	0.29	0.17	137	1.73	0.09	0.00	0.63
richness	Deviation	During Wet	(Intercept)	-0.44	0.26	308	-1.72	0.09	0.00	0.52
richness	Deviation	During Wet	trtNPK	0.21	0.12	308	1.75	0.08	0.00	0.52
richness	Deviation	One year after Dry	(Intercept)	0.11	0.21	143	0.51	0.61	0.00	0.30
richness	Deviation	One year after Dry	trtNPK	-0.01	0.17	143	-0.05	0.96	0.00	0.30
richness	Deviation	One year after Wet	(Intercept)	0.38	0.12	131	3.08	0.00	0.00	0.14
richness	Deviation	One year after Wet	trtNPK	-0.09	0.16	131	-0.57	0.57	0.00	0.14

Community aspect	Value type	Events	Terms	Value	Std. Error	DF	t-value	p	r2m	r2c
richness	Change	During Dry	(Intercept)	-0.08	0.17	344	-0.48	0.63	0.00	0.40
richness	Change	During Dry	trtNPK	0.02	0.14	344	0.13	0.90	0.00	0.40
richness	Change	During last Dry	(Intercept)	-0.29	0.29	143	-0.98	0.33	0.00	0.44
richness	Change	During last Dry	trtNPK	0.31	0.22	143	1.39	0.17	0.00	0.44
richness	Change	During last Wet	(Intercept)	-0.34	0.24	137	-1.41	0.16	0.00	0.29
richness	Change	During last Wet	trtNPK	0.21	0.26	137	0.82	0.42	0.00	0.29
richness	Change	During Wet	(Intercept)	-0.06	0.15	308	-0.41	0.68	0.00	0.30
richness	Change	During Wet	trtNPK	0.01	0.16	308	0.06	0.95	0.00	0.30
richness	Change	One year after Dry	(Intercept)	0.72	0.37	143	1.96	0.05	0.00	0.40
richness	Change	One year after Dry	trtNPK	0.09	0.28	143	0.32	0.75	0.00	0.40
richness	Change	One year after Wet	(Intercept)	0.15	0.39	131	0.38	0.70	0.00	0.48
richness	Change	One year after Wet	trtNPK	-0.06	0.27	131	-0.22	0.82	0.00	0.48
biomass	average	During normal	(Intercept)	318.55	37.95	164	8.39	0.00	0.06	0.87
biomass	average	During normal	trtNPK	152.90	12.36	164	12.37	0.00	0.06	0.87
richness	average	During normal	(Intercept)	12.18	0.67	164	18.24	0.00	0.05	0.80
richness	average	During normal	trtNPK	-2.35	0.27	164	-8.85	0.00	0.05	0.80
composition	average	During normal	(Intercept)	0.48	0.03	143	17.05	0.00	0.02	0.79
composition	average	During normal	trtNPK	-0.06	0.01	143	-4.97	0.00	0.02	0.79

Supplementary Table 5. Summaries of the estimated pairwise correlations among the five stability facets in each community aspect under control and nutrient addition treatments. Models were specified as lme (pairwise correlations ~ Treatment, random=~1|site). r2m (marginal R2): proportion of variance explained by the fixed effects in the model; r2c (conditional R2): proportion of variance explained by the fixed and random effects. SD (site): standard deviation for the random effect of sites.

Community aspect	Correlation type	Treatment	Emmean	df	lower.CL	upper.CL	r2m	r2c	SD (site)
biomass	inv_recov.d	Control	0.12	27	-0.18	0.42	0.01	0.01	0.00
biomass	inv_recov.d	NPK	-0.05	27	-0.35	0.25	0.01	0.01	0.00
biomass	inv_recov.w	Control	-0.04	30	-0.31	0.23	0.00	0.09	0.22
biomass	inv_recov.w	NPK	-0.14	30	-0.41	0.13	0.00	0.09	0.22
biomass	inv_resis.d	Control	0.29	43	0.08	0.49	0.01	0.01	0.00
biomass	inv_resis.d	NPK	0.41	43	0.20	0.61	0.01	0.01	0.00
biomass	inv_resis.w	Control	0.26	38	0.03	0.48	0.00	0.00	0.00
biomass	inv_resis.w	NPK	0.35	38	0.12	0.57	0.00	0.00	0.00
biomass	recov.d_recov.w	Control	0.15	16	-0.24	0.55	0.00	0.00	0.00
biomass	recov.d_recov.w	NPK	0.10	16	-0.30	0.49	0.00	0.00	0.00
biomass	resis.d_recov.d	Control	-0.29	27	-0.58	0.00	0.00	0.16	0.29
biomass	resis.d_recov.d	NPK	-0.25	27	-0.54	0.04	0.00	0.16	0.29
biomass	resis.d_recov.w	Control	0.03	24	-0.26	0.31	0.02	0.02	0.00
biomass	resis.d_recov.w	NPK	-0.18	24	-0.47	0.10	0.02	0.02	0.00
biomass	resis.d_resis.w	Control	0.06	27	-0.23	0.35	0.00	0.18	0.32
biomass	resis.d_resis.w	NPK	0.05	27	-0.24	0.34	0.00	0.18	0.32
biomass	resis.w_recov.d	Control	0.15	18	-0.23	0.54	0.00	0.06	0.20
biomass	resis.w_recov.d	NPK	0.11	18	-0.28	0.49	0.00	0.06	0.20
biomass	resis.w_recov.w	Control	-0.48	30	-0.72	-0.24	0.02	0.13	0.22
biomass	resis.w_recov.w	NPK	-0.28	30	-0.52	-0.04	0.02	0.13	0.22
composition	inv_recov.d	Control	-0.17	30	-0.43	0.09	0.00	0.00	0.00
composition	inv_recov.d	NPK	-0.14	30	-0.40	0.12	0.00	0.00	0.00
composition	inv_recov.w	Control	-0.14	30	-0.38	0.09	0.02	0.02	0.00
composition	inv_recov.w	NPK	-0.32	30	-0.56	-0.09	0.02	0.02	0.00
composition	inv_resis.d	Control	0.47	43	0.27	0.66	0.00	0.10	0.20
composition	inv_resis.d	NPK	0.54	43	0.35	0.74	0.00	0.10	0.20
composition	inv_resis.w	Control	0.64	38	0.46	0.82	0.00	0.04	0.12
composition	inv_resis.w	NPK	0.63	38	0.44	0.81	0.00	0.04	0.12
composition	recov.d_recov.w	Control	-0.17	16	-0.56	0.22	0.03	0.03	0.00
composition	recov.d_recov.w	NPK	0.09	16	-0.30	0.48	0.03	0.03	0.00
composition	resis.d_recov.d	Control	-0.51	30	-0.74	-0.29	0.00	0.00	0.00
composition	resis.d_recov.d	NPK	-0.51	30	-0.74	-0.29	0.00	0.00	0.00
composition	resis.d_recov.w	Control	0.11	24	-0.19	0.42	0.01	0.20	0.32
composition	resis.d_recov.w	NPK	-0.07	24	-0.37	0.24	0.01	0.20	0.32
composition	resis.d_resis.w	Control	0.14	27	-0.14	0.42	0.03	0.14	0.24
composition	resis.d_resis.w	NPK	0.41	27	0.13	0.68	0.03	0.14	0.24
composition	resis.w_recov.d	Control	-0.04	18	-0.39	0.32	0.00	0.00	0.00
composition	resis.w_recov.d	NPK	-0.12	18	-0.48	0.23	0.00	0.00	0.00
composition	resis.w_recov.w	Control	-0.42	30	-0.66	-0.17	0.01	0.08	0.18
composition	resis.w_recov.w	NPK	-0.52	30	-0.76	-0.28	0.01	0.08	0.18
richness	inv_recov.d	Control	0.07	21	-0.25	0.39	0.00	0.13	0.26
richness	inv_recov.d	NPK	0.06	21	-0.25	0.38	0.00	0.13	0.26
richness	inv_recov.w	Control	-0.20	24	-0.47	0.07	0.00	0.23	0.31
richness	inv_recov.w	NPK	-0.20	24	-0.47	0.07	0.00	0.23	0.31
richness	inv_resis.d	Control	0.31	39	0.08	0.53	0.00	0.00	0.00
richness	inv_resis.d	NPK	0.27	39	0.05	0.50	0.00	0.00	0.00
richness	inv_resis.w	Control	0.31	32	0.06	0.56	0.00	0.24	0.34
richness	inv_resis.w	NPK	0.28	32	0.03	0.53	0.00	0.24	0.34
richness	recov.d_recov.w	Control	0.32	14	-0.06	0.70	0.05	0.05	0.00
richness	recov.d_recov.w	NPK	0.03	14	-0.35	0.40	0.05	0.05	0.00
richness	resis.d_recov.d	Control	-0.16	19	-0.49	0.17	0.01	0.01	0.00
richness	resis.d_recov.d	NPK	-0.28	19	-0.61	0.05	0.01	0.01	0.00
richness	resis.d_recov.w	Control	-0.20	18	-0.51	0.12	0.04	0.06	0.10

Community aspect	Correlation type	Treatment	Emmean	df	lower.CL	upper.CL	r2m	r2c	SD (site)
richness	resis.d_recov.w	NPK	0.06	18	-0.26	0.37	0.04	0.06	0.10
richness	resis.d_resis.w	Control	-0.01	22	-0.32	0.30	0.02	0.02	0.00
richness	resis.d_resis.w	NPK	0.17	22	-0.14	0.48	0.02	0.02	0.00
richness	resis.w_recov.d	Control	0.12	15	-0.29	0.53	0.00	0.10	0.24
richness	resis.w_recov.d	NPK	0.21	15	-0.20	0.62	0.00	0.10	0.24
richness	resis.w_recov.w	Control	-0.22	21	-0.52	0.08	0.00	0.00	0.00
richness	resis.w_recov.w	NPK	-0.23	21	-0.53	0.07	0.00	0.00	0.00

Supplementary Table 6. Summaries of the estimated pairwise correlations of stability among the three community aspects under control and nutrient addition treatments. Models were specified as lme (pairwise correlations ~ Treatment, random=~1|site). r2m (marginal R2): proportion of variance explained by the fixed effects in the model; r2c (conditional R2): proportion of variance explained by the fixed and random effects. SD (site): standard deviation for the random effect of sites.

Stability facet	Correlation type	Treatment	Emmean	df	lower.CL	upper.CL	r2m	r2c	SD (site)
invariability	bio_com	Control	0.10	54	-0.09	0.29	0.03	0.03	0.00
invariability	bio_com	NPK	-0.12	54	-0.31	0.06	0.03	0.03	0.00
invariability	bio_div	Control	0.07	54	-0.12	0.26	0.00	0.00	0.00
invariability	bio_div	NPK	0.09	54	-0.10	0.29	0.00	0.00	0.00
invariability	com_div	Control	-0.03	54	-0.23	0.16	0.00	0.11	0.24
invariability	com_div	NPK	-0.08	54	-0.28	0.11	0.00	0.11	0.24
recovery_Dry	bio_com	Control	-0.02	27	-0.29	0.26	0.01	0.01	0.00
recovery_Dry	bio_com	NPK	-0.18	27	-0.46	0.09	0.01	0.01	0.00
recovery_Dry	bio_div	Control	-0.25	21	-0.58	0.08	0.00	0.00	0.00
recovery_Dry	bio_div	NPK	-0.26	21	-0.59	0.07	0.00	0.00	0.00
recovery_Dry	com_div	Control	0.29	21	-0.02	0.61	0.04	0.04	0.00
recovery_Dry	com_div	NPK	0.01	21	-0.30	0.33	0.04	0.04	0.00
recovery_Wet	bio_com	Control	-0.08	30	-0.35	0.18	0.00	0.11	0.24
recovery_Wet	bio_com	NPK	-0.01	30	-0.28	0.25	0.00	0.11	0.24
recovery_Wet	bio_div	Control	-0.13	24	-0.40	0.15	0.03	0.36	0.39
recovery_Wet	bio_div	NPK	0.11	24	-0.16	0.38	0.03	0.36	0.39
recovery_Wet	com_div	Control	0.04	24	-0.27	0.34	0.00	0.00	0.00
recovery_Wet	com_div	NPK	-0.05	24	-0.36	0.25	0.00	0.00	0.00
resistance_Dry	bio_com	Control	0.15	43	-0.07	0.37	0.02	0.09	0.19
resistance_Dry	bio_com	NPK	-0.05	43	-0.26	0.17	0.02	0.09	0.19
resistance_Dry	bio_div	Control	-0.09	39	-0.33	0.14	0.01	0.01	0.00
resistance_Dry	bio_div	NPK	0.03	39	-0.20	0.27	0.01	0.01	0.00
resistance_Dry	com_div	Control	0.08	39	-0.14	0.30	0.01	0.01	0.00
resistance_Dry	com_div	NPK	-0.05	39	-0.27	0.17	0.01	0.01	0.00
resistance_Wet	bio_com	Control	0.03	38	-0.20	0.27	0.01	0.01	0.00
resistance_Wet	bio_com	NPK	-0.13	38	-0.36	0.10	0.01	0.01	0.00
resistance_Wet	bio_div	Control	0.14	32	-0.11	0.40	0.01	0.01	0.00
resistance_Wet	bio_div	NPK	0.02	32	-0.24	0.27	0.01	0.01	0.00
resistance_Wet	com_div	Control	0.00	32	-0.26	0.25	0.00	0.00	0.00
resistance_Wet	com_div	NPK	-0.10	32	-0.35	0.15	0.00	0.00	0.00

Supplementary Table 7. Principal investigators who contribute data are not authors; site names match those in Supplementary Table 1. Their effort in providing data is critical to this manuscript.

site_code	PI.name	Institution
arch.us	Elizabeth Boughton	MacArthur Agro-ecology Research Center
badlau.de	Sylvia Haider	Martin-Luther-UniversitÃ¤t Halle-Wittenberg
badlau.de	Julia Siebert	German Centre for Integrative Biodiversity Research (iDiv)
bayr.de	Marie Spohn	NULL
bogong.au	Joslin Moore	University of Melbourne
burrawan.au	Jennifer Firn	Queensland University of Technology
cbgb.us	Lori Biederman	Iowa State University
cbgb.us	Kirsten Hofmockel	Iowa State University
cbgb.us	Lauren Sullivan	Iowa State University
cdcr.us	Adam Kay	University of St. Thomas
cdpt.us	Johannes Knops	University of Nebraska, Lincoln
chilcas.ar	Enrique Chaneton	Universidad de Buenos Aires
chilcas.ar	Laura Yahdjian	Universidad de Buenos Aires
elliot.us	Elsa Cleland	University of California, San Diego
frue.ch	Sabine GÃ¼sewell	ETH Zurich
frue.ch	Andy Hector	University of Zurich
hall.us	Rebecca McCulley	University of Kentucky
hall.us	Jim Nelson	University of Kentucky
hart.us	Nicole DeCrappeo	USGS
hart.us	David Pyke	USGS
hero.uk	Mick Crawley	Imperial College at Silwood Park
kbs.us	Lars Brudvig	Michigan State University
koffler.ca	Marc Cadotte	University of Toronto Scarborough
koffler.ca	Arthur Weiss	University of Toronto Scarborough
konz.us	Kimberly Komatsu	University of California, Berkeley
konz.us	Melinda Smith	Colorado State University
msla.us	Kelly Laflamme	MPG Ranch
mtca.au	Suzanne Prober	CSIRO
ping.au	Jodi Price	The University of Western Australia
ping.au	Rachel Standish	The University of Western Australia
potrok.ar	Hector Bahamonde	UNPA - CONICET
potrok.ar	Pablo Peri	UNPA - CONICET
rook.uk	Mick Crawley	Imperial College at Silwood Park
sage.us	Louie Yang	University of California, Davis
saline.us	Kimberly Komatsu	University of California, Berkeley
saline.us	Melinda Smith	Colorado State University
sedg.us	Carla D'Antonio	University of California, Santa Barbara
sevi.us	Scott Collins	University of New Mexico
sevi.us	Laura Ladwig	University of New Mexico
sgs.us	Dana Blumenthal	USDA-ARS
sgs.us	Cynthia Brown	Colorado State University
sgs.us	Julia Klein	Colorado State University
sgs.us	Alan Knapp	Colorado State University
shps.us	Peter Adler	Utah State University
smith.us	Janneke Hille Ris Lambers	University of Washington
spin.us	Rebecca McCulley	University of Kentucky
spin.us	Jim Nelson	University of Kentucky
temple.us	Philip Fay	USDA-ARS
trel.us	Andrew Leakey	University of Illinois at Urbana-Champaign
ukul.za	Kevin Kirkman	University of KwaZulu-Natal
ukul.za	Michelle Tedder	University of KwaZulu-Natal
unc.us	Charles Mitchell	University of North Carolina
unc.us	Justin Wright	Duke University
valm.ch	Martin Schuetz	Swiss Federal Institute for Forest, Snow and Landscape Research
yarra.au	Raul Ochoa Hueso	University of Western Sydney

Supplementary Table 8. Contributions of each author to the manuscript.

Full name	Site(s) used in analysis	Developed and framed research question(s)	Analyzed data	Contributed to data analyses	Wrote the paper	Contributed to paper writing	Site coordinator	Nutrient Network coordinator	Site-level acknowledgments (funding, access, etc)
Qingqing Chen	NA	x	x	NA	x	NA	NA	NA	NA
Yann Hautier	Frue.ch	x	x	NA	NA	x	x	NA	NA
Shaopeng Wang	NA	x	x	NA	NA	x	NA	NA	NA
Johannes M H Knops	cdpt	NA	NA	NA	NA	x	x	NA	NA
Anita C. Risch	valm.ch	NA	NA	NA	NA	x	x	NA	NA
Eric W. Seabloom	bnch.us, cdcr.us, hopl.us, look.us, mcla.us, sier.us	NA	NA	NA	NA	x	x	x	NA
Anne Ebeling	jena.de	NA	NA	NA	NA	x	x	NA	NA
John W. Morgan	bogong.au, kiny.au	NA	NA	NA	NA	x	x	NA	NA
Christiane Roscher	jena.de	NA	NA	NA	NA	x	x	NA	NA
Sally A. Power	NA	NA	NA	NA	NA	x	x	NA	NA
Jason P. Martina	temple.us	NA	NA	NA	NA	x	x	NA	NA
Jane A. Catford	nilla.au	NA	NA	NA	NA	X	X	NA	NA
Maria C. Caldeira	comp.pt	NA	NA	NA	NA	x	x	NA	Portuguese Science Foundation (FCT) for funding the research unit CEF (UIDB/00239/2020) and to Companhia das Lezirias for field access
Miguel N Bugalho	comp.pt	NA	NA	NA	NA	x	x	NA	Portuguese Science Foundation (FCT) for funding the research unit CEABN-InBIO (UIDB/50027/2020) and to Rui Alves for logistic suport and granting access to study site

Full name	Site(s) used in analysis	Developed and framed research question(s)	Analyzed data	Contributed to data analyses	Wrote the paper	Contributed to paper writing	Site coordinator	Nutrient Network coordinator	Site-level acknowledgments (funding, access, etc)
Elizabeth T Borer	bnch.us, cdcr.us, hopl.us, look.us, mcla.us, sier.us	NA	NA	NA	NA	x	x	x	NA
Risto Virtanen	kilp.fi, saana.fi	NA	NA	NA	NA	x	x	NA	NA
Anu Eskelinen	kilp.fi, saana.fi	NA	NA	NA	NA	x	x	NA	NA
Nico Eisenhauer	badlau.de	NA	NA	NA	NA	x	x	NA	Further support came from the German Centre for Integrative Biodiversity Research (iDiv) Halle-Jena-Leipzig, funded by the German Research Foundation (FZT 118, 202548816).
W Stanley Harpole	mcla.us, sier.us	NA	NA	NA	NA	x	x	NA	Further support came from the German Centre for Integrative Biodiversity Research (iDiv) Halle-Jena-Leipzig, funded by the German Research Foundation (FZT 118, 202548816).
Ian Donohue	burren.ie	NA	NA	NA	NA	x	x	NA	National Parks and Wildlife Service, Ireland
Yvonne M. Buckley	burren.ie	NA	NA	NA	NA	x	x	NA	National Parks and Wildlife Service, Ireland
Isabel C Barrio	ahth.is, amlr.is	NA	NA	NA	NA	x	x	NA	University of Iceland Research Fund (2015), Soil Conservation Service of Iceland, Orkurannsóknasjóður Landsvirkjunnar (NÝR-09-2017, NÝR-14-2018, NÝR-12-2019)

Full name	Site(s) used in analysis	Developed and framed research question(s)	Analyzed data	Contributed to data analyses	Wrote the paper	Contributed to paper writing	Site coordinator	Nutrient Network coordinator	Site-level acknowledgments (funding, access, etc)
Jonathan D. Bakker	smith.us	NA	NA	x	NA	x	x	NA	NA
Anke Jentsch	bayr.de	NA	NA	NA	NA	x	x	NA	Federal Ministry of Education and Research BMBF (FKZ 031B0516C, 031B1067C)
Yujie Niu	bayr.de	NA	NA	double checking the code	NA	x	NA	NA	Y. Niu is a Humboldt fellow funded by Alexander von Humboldt-Stiftung.
Juan Alberti	marc.ar	NA	NA	x	NA	x	NA	NA	NA
Pedro Daleo	marc.ar	NA	NA	x	NA	x	NA	NA	NA
Carly J. Stevens	lancaster.uk	NA	NA	NA	NA	x	x	NA	NA
Ylva Lekberg	msla_us; msla_2us; msla_3us	NA	NA	NA	NA	x	x	NA	MPG Ranch
Daniel S Gruner	sage.us	NA	NA	NA	NA	x	x	NA	NA
Andrew MacDougall	Cowi.ca	NA	NA	NA	NA	x	x	NA	NA

Supplementary Notes

Normal levels, change and deviation in community aspects On average, nutrient addition led to a 48% increase in aboveground biomass during normal growing seasons. Nutrient addition significantly decreased aboveground biomass during dry growing seasons while weakly increasing it during wet growing seasons. But it significantly increased biomass deviation during both dry and wet growing seasons from the normal levels (note, change can be positive or negative values; while deviation is always positive values; Supplementary Figure 7). Nutrient addition also significantly increased biomass deviation one year after the dry growing seasons. Nutrient addition significantly decreased community similarity during normal growing seasons, during and after both dry and wet growing seasons relative to that of normal levels (Supplementary Figure 8). Nutrient addition decreased species richness by 19% during normal growing seasons. Nutrient addition had no effects on change in species richness during dry and wet seasons from the normal levels (Supplementary Figure 9). However, nutrient addition significantly increased deviation in species richness during dry growing seasons, suggesting some sites increased while other sites decreased in species richness during dry growing seasons (relative to their normal levels). Nutrient addition had no effects on deviation in species richness during wet growing seasons, suggesting most sites did not strongly change species richness during wet growing seasons from their normal levels (Supplementary Figure 9).
