# Supplementary material for: Multidimensional responses of grassland stability to eutrophication
Source: Nat Commun. 2023 Oct 11;14:6375. doi: 10.1038/s41467-023-42081-0 (PMC10567679; doi:10.1038/s41467-023-42081-0)
Supplement: Supplementary file 2 — Reporting Summary [file 41467_2023_42081_MOESM2_ESM.pdf]

nature portfolio

Corresponding author(s): Shaopeng Wang

Last updated by author(s): 29-08-2023

Reporting Summary

Nature Portfolio wishes to improve the reproducibility of the work that we publish. This form provides structure for consistency and transparency in reporting. For further information on Nature Portfolio policies, see our Editorial Policies and the Editorial Policy Checklist.

Statistics

For all statistical analyses, confirm that the following items are present in the figure legend, table legend, main text, or Methods section.

n/a	Confirmed	
☐	☒	The exact sample size (*n*) for each experimental group/condition, given as a discrete number and unit of measurement
☐	☒	A statement on whether measurements were taken from distinct samples or whether the same sample was measured repeatedly
☐	☒	The statistical test(s) used AND whether they are one- or two-sided<br>*Only common tests should be described solely by name; describe more complex techniques in the Methods section.*
☐	☒	A description of all covariates tested
☐	☒	A description of any assumptions or corrections, such as tests of normality and adjustment for multiple comparisons
☐	☒	A full description of the statistical parameters including central tendency (e.g. means) or other basic estimates (e.g. regression coefficient) AND variation (e.g. standard deviation) or associated estimates of uncertainty (e.g. confidence intervals)
☐	☒	For null hypothesis testing, the test statistic (e.g. *F*, *t*, *r*) with confidence intervals, effect sizes, degrees of freedom and *P* value noted<br>*Give P values as exact values whenever suitable.*
☒	☐	For Bayesian analysis, information on the choice of priors and Markov chain Monte Carlo settings
☐	☒	For hierarchical and complex designs, identification of the appropriate level for tests and full reporting of outcomes
☐	☒	Estimates of effect sizes (e.g. Cohen's *d*, Pearson's *r*), indicating how they were calculated

Our web collection on statistics for biologists contains articles on many of the points above.

Software and code

Policy information about availability of computer code

Data collection	Temporal invariability of aboveground biomass and species richness was calculated after detrending to remove variation due to directional change over time. That is, we first used linear regression (function “lm” from base R) to fit species richness or biomass against experimental years for each subplot, we then used the residuals from this model to calculate the standard deviation. To enable comparison among sites with varying conditions, we quantified resistance as the inverse of the proportional deviation of a community aspect from its normal levels during a dry or wet growing season. We quantified recovery as the ratio of deviation in a community aspect during to that after a dry or wet growing season. The composition-related facets of stability were calculated using Bray–Curtis dissimilarity metric based on cover data. Temporal invariability was calculated as the overall community similarity over all experimental years using the function “beta.multi.abund” from the R package betapart (version 1.6). The average cover for all species during normal growing seasons under each treatment within a block was constructed as a reference community for calculating resistance and recovery. Resistance was calculated as the similarity of the plant community under an extreme growing season compared with the reference using the R function “beta.pair.abund”. Similarly, we calculated similarity of the plant community one year after an extreme growing season compared with the reference. Recovery was then calculated as the ratio of similarity of the community one year after to that during an extreme growing season.<br>The R codes used to produce results in this study have been deposited in the GitHub (https://github.com/chqq365/multidimensional-responses-of-stability.git.) and archived through Zenodo (https://doi.org/10.5281/zenodo.8292710).
Data analysis	All analyses were performed in R (version 4.2.0). We used linear mixed-effects models (function “lme”) from the R package nlme (version 3.1.157) for all statistic tests.

For manuscripts utilizing custom algorithms or software that are central to the research but not yet described in published literature, software must be made available to editors and reviewers. We strongly encourage code deposition in a community repository (e.g. GitHub). See the Nature Portfolio guidelines for submitting code & software for further information.

Data

Policy information about availability of data

All manuscripts must include a data availability statement. This statement should provide the following information, where applicable:

- Accession codes, unique identifiers, or web links for publicly available datasets
- A description of any restrictions on data availability
- For clinical datasets or third party data, please ensure that the statement adheres to our policy

We used experimental data from 55 grassland sites that are part of the Nutrient Network (NutNet) Global Research Cooperative. See http://www.nutnet.org for more details for this network. Sites cover 5 continents and 12 countries, and a wide range of grassland types. For the analyses here, we select plots assigned to one of two treatments: Control or Fertilized (NPK+µ). 5 m × 5 m plots were assigned to one of the two treatments in a randomized block design, typically with three blocks per site. The raw data used and processed data generated in this study have been deposited in the Figshare (https://doi.org/10.6084/m9.figshare.22639399).

Research involving human participants, their data, or biological material

Policy information about studies with human participants or human data. See also policy information about sex, gender (identity/presentation), and sexual orientation and race, ethnicity and racism.

Reporting on sex and gender	N/A
Reporting on race, ethnicity, or other socially relevant groupings	N/A
Population characteristics	N/A
Recruitment	N/A
Ethics oversight	N/A

Note that full information on the approval of the study protocol must also be provided in the manuscript.

Field-specific reporting

Please select the one below that is the best fit for your research. If you are not sure, read the appropriate sections before making your selection.

☐ Life sciences ☐ Behavioural & social sciences ☒ Ecological, evolutionary & environmental sciences

For a reference copy of the document with all sections, see nature.com/documents/nr-reporting-summary-flat.pdf

Ecological, evolutionary & environmental sciences study design

All studies must disclose on these points even when the disclosure is negative.

Study description	The 55 study sites are part of the NutNet experiment. For the analyses here, we select plots assigned to one of two treatments: Control or Fertilized (NPK+µ). 5 m × 5 m plots were assigned to one of the two treatments in a randomized block design, typically with three blocks per site. NPK+µ treatment plots were fertilized with nitrogen (N), phosphorus (P), and potassium with a combination of micronutrients and macronutrients (Fe, S, Mg, Mn, Cu, Zn, B, and Mo) as a one-time addition to the potassium treatment (K+µ). The micronutrient mix was applied once at the start of the experiment at a rate of 100 g m-2. N was supplied as time-release urea ((NH2)2CO), P was supplied as triple superphosphate (Ca(H2PO4)2), and K as potassium sulfate (K2SO4). N, P, and K were added annually at rates of 10 g m-2 y-1.
Research sample	Site PIs recorded plant aboveground biomass, percent cover for individual species, and species richness from 55 grassland sites across the globe (https://nutnet.org/).
Sampling strategy	A 1 ×1 m subplot within each plot was permanently marked for annual measurement of plant community composition. Sampling was done in 1 m2 subplots and followed a standardized protocol at all sites. The number of samples was chosen to balance the replicates needed for statistics and the efforts needed for sampling at each site.
Data collection	Data collection was done by the principal investigator at each site. All NutNet sites followed standard sampling protocols, detailed in (Borer, E. T. et al. Finding generality in ecology: a model for globally distributed experiments. Methods in Ecology and Evolution 5, 63-73 (2013)). Species cover (%) was estimated visually for all species in the subplots; the total cover of living plants can exceed 100 % for multilayer canopies. Aboveground biomass was measured within the treatment plot, adjacent to the permanent subplot, by clipping all aboveground biomass within two 1 × 0.1 m strips (in total 0.2 m2), which were moved each year to avoid resampling the same location. For shrubs and subshrubs occurring in strips, we collected all leaves and current year's woody growth. Biomass was dried at

60 °C (to constant mass) before weighing to the nearest 0.01 g, and expressed as g m-2.

Timing and spatial scale

At most sites, cover was recorded once per year at peak biomass before fertilization (peak biomass season was estimated by site PIs). At some sites with strong seasonality, cover was recorded twice per year to include a complete list of species. For those sites, the maximum cover for each species and total biomass were used in the following analyses. Sampling was done in 1 m2 subplots and followed a standardized protocol at all sites. subplots were nested within blocks. A block typically spreads over 320 m2, and all three blocks typically spread over >1000 m2.

Data exclusions

Exclusion criteria
were pre-established. The 55 sites included in this study met the following criteria: (1) plots were arranged in 3 blocks; (2) ≥ 4 years of post-treatment measurement; (3) during experimental years, at least one dry or wet growing season occurred (see "Defining climate extremes" for more details). These sites span five continents and include a wide range of grassland types.

Reproducibility

To increase transparency and reproducibility, the raw data used and processed data generated in this study have been deposited in the Figshare (https://doi.org/10.6084/m9.figshare.22639399). The R codes used to produce results in this study have been deposited in the GitHub (https://github.com/chqq365/multidimensional-responses-of-stability.git.) and archived through Zenodo (https://doi.org/10.5281/zenodo.8292710).

Randomization

Blocks within sites were randomly assigned. Treatments were randomly assigned.

Blinding

The site PIs know the location for each treatment at each site. However, most site PIs did not directly involve in data analyses. Data were analyzed using all sites together, it is difficult to know which site had which influence on the overall results.

Did the study involve field work? ☒ Yes ☐ No

Field work, collection and transport

Field conditions

Our 55 sites were distributed over 5 continents and 12 countries. All sites are dominated by herbaceous species. Sites cover a wide range of grassland types that range from alpine grassland, to prairie, pasture, shrub steppe, savanna and old field. Sites also cover a wide range of climate condition (e.g. latitude, longitude), and water balance (taking into account both temperature and rainfall). See below for details.

Location

site_code; habitat; continent; latitude; longitude; first experimental year; growing season water balance during last 15 years
ahth.is heathland Europe 65.13 -19.67 2016 6-9 57.72
amlr.is desert grassland Europe 65.13 -19.67 2016 6-9 57.72
arch.us mixedgrass prairie North America 27.17 -81.22 2016 5-10 177.28
badlau.de old field Europe 51.39 11.88 2016 4-10 -263.08
bayr.de mesic grassland Europe 49.92 11.58 2017 3-9 -202.68
bnch.us montane grassland North America 44.28 -121.97 2008 4-8 -410.42
bogong.au alpine grassland Australia -36.87 147.25 2010 10-1 -161.27
burrawan.au semiarid grassland Australia -27.74 151.14 2009 10-5 -768.43
burren.ie calcareous grassland Europe 53.07 -8.99 2016 2-8 169.66
cbgb.us tallgrass prairie North America 41.79 -93.39 2010 5-10 -121.7
cdcr.us tallgrass prairie North America 45.42 -93.21 2008 4-8 -198.09
cdpt.us shortgrass prairie North America 41.21 -101.64 2008 4-7 -320.66
chilcas.ar mesic grassland South America -36.28 -58.27 2014 8-3 -283.02
comp.pt annual grassland Europe 38.83 -8.79 2013 10-5 26.15
cowi.ca old field North America 48.81 -123.63 2008 4-7 -126.39
elliot.us annual grassland North America 32.88 -117.05 2009 11-4 -320.77
frue.ch pasture Europe 47.11 8.54 2009 4-9 200.73
hall.us tallgrass prairie North America 36.87 -86.7 2008 4-9 -146.23
hart.us shrub steppe North America 42.72 -119.5 2008 10-7 -663.66
hero.uk mesic grassland Europe 51.41 -0.64 2008 4-10 -114.17
hopl.us annual grassland North America 39.01 -123.06 2008 11-4 519.56
jena.de grassland Europe 50.94 11.53 2014 3-10 -179.13
kbs.us old field North America 42.41 -85.39 2014 4-9 -198.2
kilp.fi tundra grassland Europe 69.06 20.87 2014 6-9 24.72
kiny.au semiarid grassland Australia -36.2 143.75 2008 5-10 -201.76
koffler.ca pasture North America 44.02 -79.54 2011 4-8 -160.54
konz.us tallgrass prairie North America 39.07 -96.58 2008 5-9 -233.79
lancaster.uk mesic grassland Europe 53.99 -2.63 2009 3-8 76.77
look.us montane grassland North America 44.21 -122.13 2008 3-8 -205.99
marc.ar grassland South America -37.72 -57.42 2012 4-12 -39.35
mcla.us annual grassland North America 38.86 -122.41 2008 11-4 271.61
msla.us grassland North America 46.66 -114 2018 4-7 -389.77
msla_2.us grassland North America 46.66 -114 2018 4-7 -779.53
msla_3.us grassland North America 46.66 -114 2018 4-7 -779.53
mtca.au savanna Australia -31.78 117.61 2009 8-10 -261.25
nilla.au old field Australia -36.9 146.01 2017 2-1 -288.9

ping.au old field Australia -32.5 116.97 2014 4-10 -230.83
potrok.ar semiarid grassland South America -51.92 -70.41 2016 10-4 -581.11
rook.uk mesic grassland Europe 51.41 -0.64 2008 4-10 -114.17
saana.fi montane grassland Europe 69.04 20.84 2015 6-9 24.72
sage.us montane grassland North America 39.43 -120.24 2008 4-7 -466.61
saline.us mixedgrass prairie North America 39.05 -99.1 2008 5-9 -482.76
sedg.us annual grassland North America 34.7 -120.02 2008 11-7 -459.86
sevi.us desert grassland North America 34.36 -106.69 2008 4-11 -1214.75
sgs.us shortgrass prairie North America 40.82 -104.77 2008 4-8 -565.4
shps.us shrub steppe North America 44.26 -112.21 2008 4-9 -698.95
sier.us annual grassland North America 39.24 -121.28 2008 11-4 415.05
smith.us mesic grassland North America 48.21 -122.62 2008 10-6 130.83
spin.us pasture North America 38.13 -84.5 2008 3-5 47.75
temple.us tallgrass prairie North America 31.04 -97.35 2008 3-10 -656.61
trel.us tallgrass prairie North America 40.08 -88.83 2009 4-9 -166.75
ukul.za mesic grassland Africa -29.67 30.4 2010 9-4 -118.91
unc.us old field North America 36.01 -79.02 2008 4-9 -195.07
valm.ch alpine grassland Europe 46.63 10.37 2009 6-8 176.95
yarra.au mesic grassland Australia -33.61 150.74 2015 9-3 -407.76

Access & import/export: Access to sites and data collection followed standard practices and complies with laws. No permits were needed.

Disturbance: Walkways were established to minimize trampling effects from site PIs on the sampling plots.

Reporting for specific materials, systems and methods

We require information from authors about some types of materials, experimental systems and methods used in many studies. Here, indicate whether each material, system or method listed is relevant to your study. If you are not sure if a list item applies to your research, read the appropriate section before selecting a response.

Materials & experimental systems

n/a	Involved in the study
☒	☐ Antibodies
☒	☐ Eukaryotic cell lines
☒	☐ Palaeontology and archaeology
☒	☐ Animals and other organisms
☒	☐ Clinical data
☒	☐ Dual use research of concern
☐	☒ Plants

Methods

n/a	Involved in the study
☒	☐ ChIP-seq
☒	☐ Flow cytometry
☒	☐ MRI-based neuroimaging

Dual use research of concern

Policy information about dual use research of concern

Hazards

Could the accidental, deliberate or reckless misuse of agents or technologies generated in the work, or the application of information presented in the manuscript, pose a threat to:

No	Yes
☒	☐ Public health
☒	☐ National security
☒	☐ Crops and/or livestock
☒	☐ Ecosystems
☒	☐ Any other significant area

Experiments of concern

Does the work involve any of these experiments of concern:

No	Yes	
☒	☐	Demonstrate how to render a vaccine ineffective
☒	☐	Confer resistance to therapeutically useful antibiotics or antiviral agents
☒	☐	Enhance the virulence of a pathogen or render a nonpathogen virulent
☒	☐	Increase transmissibility of a pathogen
☒	☐	Alter the host range of a pathogen
☒	☐	Enable evasion of diagnostic/detection modalities
☒	☐	Enable the weaponization of a biological agent or toxin
☒	☐	Any other potentially harmful combination of experiments and agents
